# Cavity-based lymphomas: challenges and novel concepts. A report of the 2022 EA4HP/SH lymphoma workshop

**DOI:** 10.1007/s00428-023-03599-2

**Published:** 2023-08-09

**Authors:** Arianna Di Napoli, Lori Soma, Leticia Quintanilla-Martinez, Laurence de Leval, Lorenzo Leoncini, Alberto Zamò, Siok-Bian Ng, Sarah L. Ondrejka, Fina Climent, Andrew Wotherspoon, Stefan Dirnhofer

**Affiliations:** 1 Department of Clinical and Molecular Medicine, Sant’Andrea University Hospital, Sapienza University of Rome, Rome, Italy; 2 Department of Pathology, City of Hope National Medical Center, Duarte, CA USA; 3 Institute of Pathology and Neuropathology, Eberhard Karls University of Tübingen and Comprehensive Cancer Center, University Hospital Tübingen, Tübingen, Germany; 4 Institute of Pathology, Department of Laboratory Medicine and Pathology, Lausanne University Hospital and Lausanne University, Lausanne, Switzerland; 5 Department of Medical Biotechnology, University of Siena, Siena, Italy; 6 Institute of Pathology, University of Würzburg, Würzburg, Germany; 7 Department of Pathology, Yong Loo Lin School of Medicine, National University of Singapore, Singapore, Singapore; 8 Pathology, and Laboratory Medicine Institute, Cleveland Clinic, Cleveland, OH USA; 9 Pathology Department, Hospital Universitari de Bellvitge, IDIBELL, L’Hospitalet De Llobregat, Barcelona, Spain; 10 Department of Histopathology, The Royal Marsden Hospital, London, UK; 11 Institute of Medical Genetics and Pathology, University Hospital Basel, University of Basel, Basel, Switzerland

**Keywords:** Pleural Effusion Lymphoma, Extracavitary primary effusion lymphoma, Germinotropic disorder, Fluid overload associated large B cell lymphoma, Effusion-based large B cell lymphoma, HHV8-negative effusion lymphoma, Fibrin-associated large B cell lymphoma, Breast Implant associated anaplastic large cell lymphoma

## Abstract

The 2022 European Association for Haematopathology/Society for Hematopathology lymphoma workshop session on cavity-based lymphomas included sixty-eight cases in seven sections. The disease entities discussed include primary effusion lymphomas (PEL), extracavitary primary effusion lymphomas and confounding entities (ECPEL), HHV8-negative B-lineage lymphomas-effusion based (EBV-negative, EBV-positive, and plasmablastic types), diffuse large B-cell lymphoma associated with chronic inflammation, fibrin-associated diffuse large B-cell lymphoma (FA-DLBCL), breast implant-associated anaplastic large cell lymphoma (BIA-ALCL), and other lymphomas presenting as an effusion. All entities above are discussed; however, three are delved into greater detail given the challenges with classification: ECPEL, HHV8-negative effusion-based lymphomas, and FA-DLBCL. Cases exemplifying the diagnostic difficulty in differentiating ECPEL from HHV8-positive diffuse large B-cell lymphoma and germinotropic lymphoproliferative disorder were discussed. The more recently recognized effusion-based HHV8-negative large B-cell lymphoma is explored, with several cases submitted raising the question if this subset should be carved out as a specific entity, and if so, what should be the refining diagnostic criteria. Case submissions to the FA-DLBCL section yielded one of the largest case series to date, including classic cases, cases furthering the discussion on disease sites and prognosis, as well as novel concepts to be considered in this entity. The 2022 EA4HP/SH workshop cases allowed for further confirmation of the characteristics of some of the more historically accepted cavity-based lymphomas, as well as further inquiry and debate on relatively new or evolving entities.

**Supplementary Information:**

The online version contains supplementary material available at 10.1007/s00428-023-03599-2.

## Introduction

The 2022 European Association of Haematopathology/Society for Hematopathology Lymphoma Workshop (EA4HP/SH LW) was held in Florence, Italy. The second session of the lymphoma workshop was a discussion of cavity-based lymphomas chaired by S. Dirnhofer, L. Soma, and A. Di Napoli.

Sixty-eight cases were submitted to this section (submitted case data in Supplementary tables), and according to the WHO revised 4th edition (WHO-4R), it included primary effusion lymphoma/extracavitary primary effusion lymphoma (PEL/ECPEL), HHV8-negative effusion-based lymphomas (mentioned in the PEL chapter but not described as a specific entity), diffuse large B-cell lymphoma associated with chronic inflammation (CI-DLBCL – to include fibrin-associated large B-cell lymphoma, a subcategory of CI-DLBCL), and breast implant-associated anaplastic large cell lymphoma (provisional entity) [1]. The cases submitted raised additional questions on challenging classifications, separating subcategories out as defined entities, and differential diagnoses that need to be considered in unusual settings. Of note, some of these entities were previously discussed in recent workshops (2015 SH/EA4HP, 2018 EA4HP/SH, and 2019 Chinese Society for Hematopathology/SH Workshops) [2, 3].

## Primary effusion lymphoma (PEL)

PEL is a diagnostic entity in the WHO-4R and recognized in both the 5th edition WHO manuscript (WHO-5) [4] and International Consensus Classification (ICC) [5], as a neoplasm universally associated with Kaposi sarcoma herpes virus/human herpes virus 8 (KSHV/HHV8), which is immunohistochemically positive for HHV8 latent nuclear antigen-1 (LANA-1). It is effusion-based, usually without a solid component (although extracavitary primary effusion lymphoma, ECPEL, is also included). The most common settings for these lymphomas are male, human immunodeficiency virus (HIV) positive (median age 43) or elderly patients (median age 73), and association with multicentric Castleman disease (MCD) and/or Kaposi sarcoma [1]. While KSHV/HHV8 is a necessary factor for lymphomagenesis, it is not sufficient on its own, and lymphoma development relies on additional factors [6, 7]. PEL can either be Epstein-Barr virus (EBV) positive or negative, with the majority of EBV-positive cases involving severely immunocompromised HIV-positive men and the EBV-negative cases typically occurring in HIV-negative elderly men. Interestingly, post-transplant PEL is more often EBV-negative (the EA4HP cohort confirmed these findings) [8, 9]. Morphologically, the neoplasm is composed of large cells with plasmablastic/immunoblastic/anaplastic cytology and with a terminal B-lineage immunophenotype, positive for CD30, CD38, CD138, EMA, MUM1, and HLA-DR and typically absent for PAX5, CD19, CD20, and CD79a. CD45 is usually expressed, and although not common, expression of T/NK markers may occur (more so in ECPEL, where expressions of CD20 and CD79a are also more frequent and CD45 less common). Immunoglobulin heavy chain (IGH) gene is clonally rearranged and hypermutated, with a subset also having clonal T-cell receptor (TR) gene rearrangement. No specific chromosomal abnormalities are present; however, the karyotype is often complex and without *MYC* rearrangement (although extra copies may be present) [1, 10]. Mutations in *BCL6, MYC, PAX5*, and *RhoH/TTF* have been reported, with a lack of *TP53* and *RAS* family mutations [1, 7]. Gene expression profiling (mostly in cell lines) has shown that PELs are reminiscent of plasmablastic lymphomas, demonstrating an expression profile of plasma cells/myeloma, as well as immunoblasts/diffuse large B-cell lymphoma, with differential expression profiles noted between EBV-positive and EBV-negative PELs [7, 11–13]. Reported median overall survival has ranged from 6 to 42.5 months, with antiretroviral therapy being an important component in achieving better survival rates [7, 10].

**Fig. 1:**
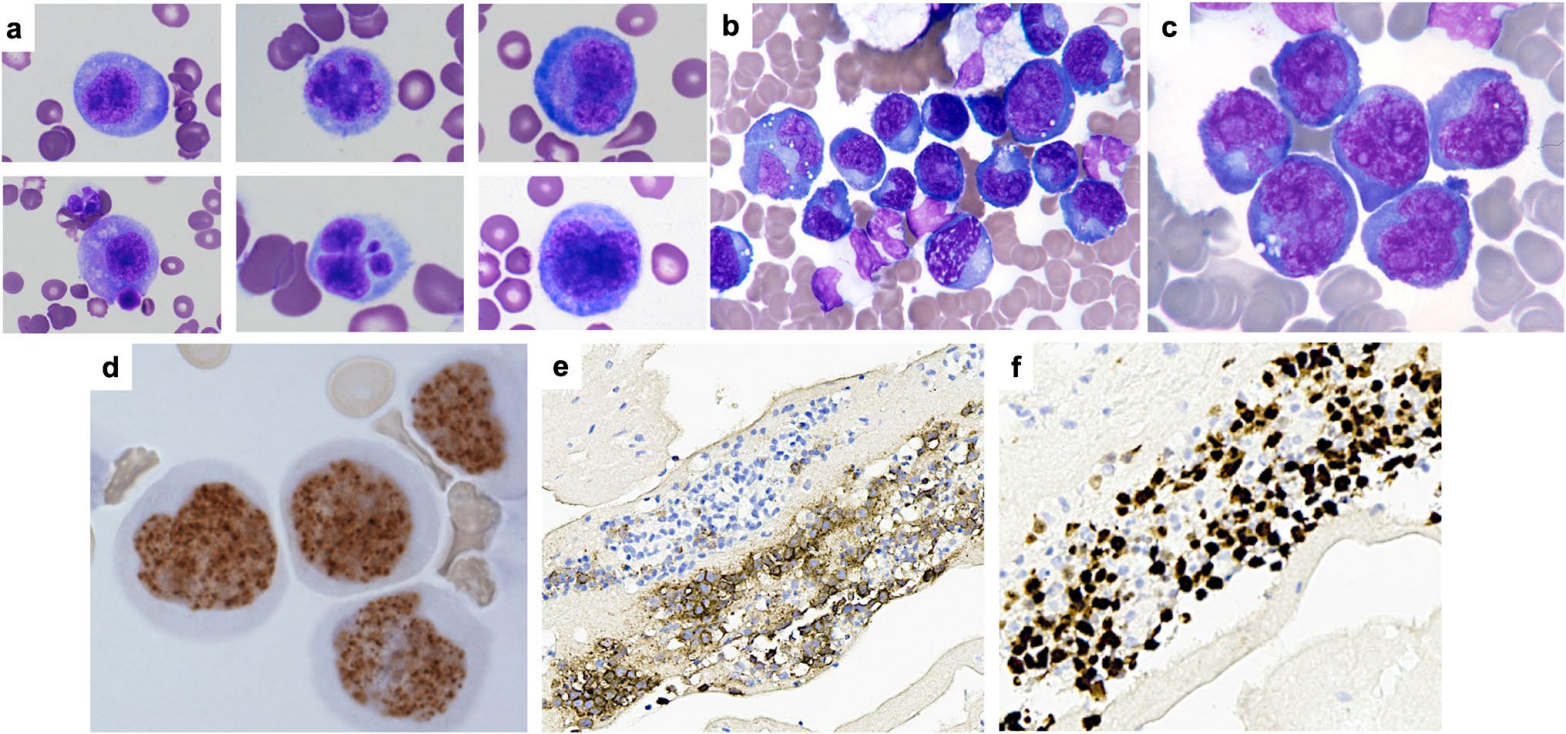
Histologic and immunophenotypic features of primary effusion lymphoma. (**a**–**c)** Smears from plural effusions demonstrating plasmablastic, immunoblastic, and anaplastic morphology seen in an HIV-positive, EBV-positive patient (**a**. LYWS-1050 courtesy of A. Shestakov) and an HIV-negative, EBV-negative patient (**b**–**f**. LYWS-1194 courtesy of P. Barone). (**d**) LANA (KSHV/HHV8) positivity, demonstrating the typical speckled pattern. (**e**–**f**) CD138 (**e**) and MUM1 (**f**) expression in neoplastic cells

**Fig. 2:**
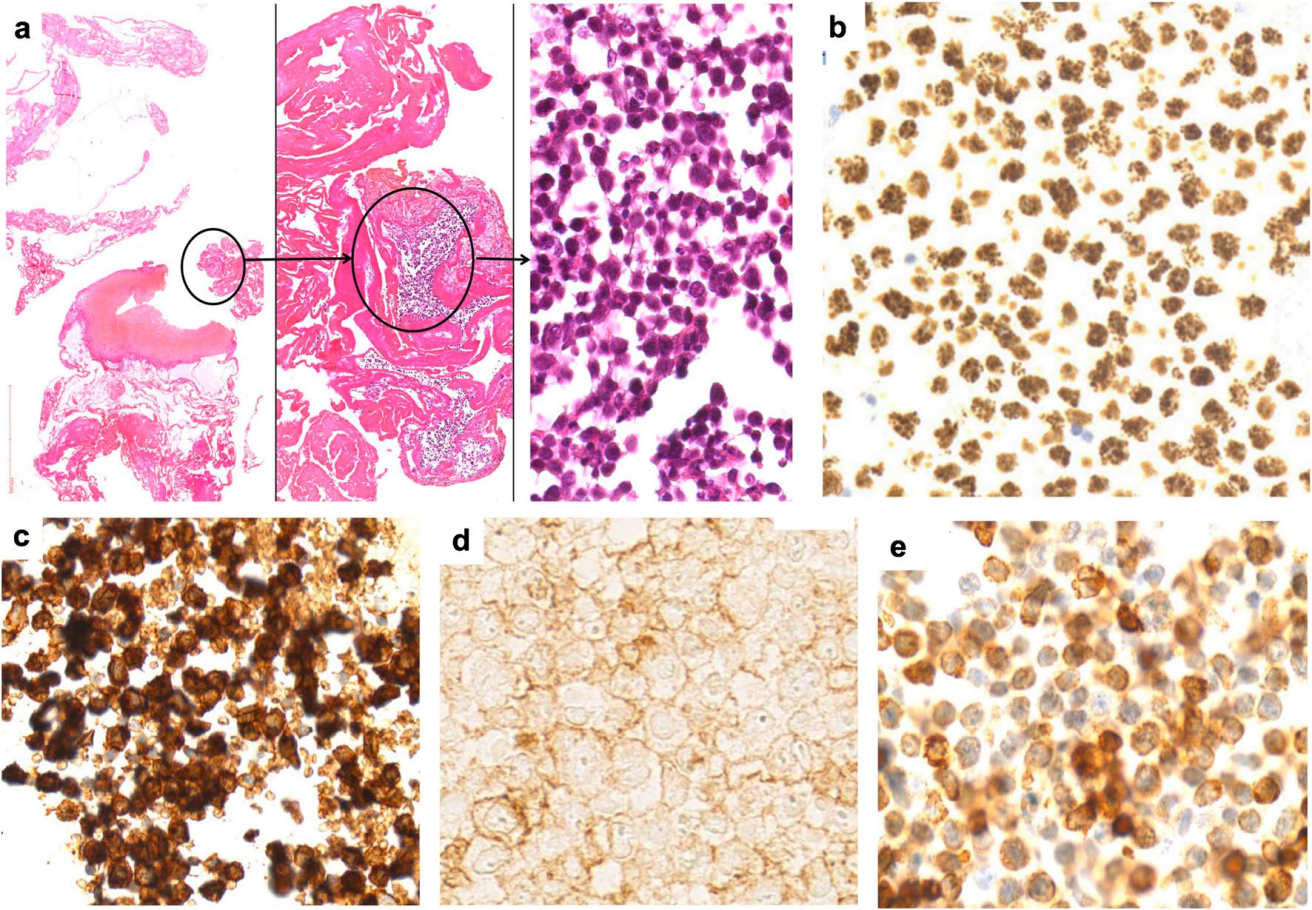
Example of primary effusion lymphoma with expression of T-cell markers (LYWS-1216 courtesy of L. Mescam). (**a**) Histologic sections from the peritoneal biopsy demonstrating neoplastic cells with immunoblastic cytomorphology within fibrinous material. (**b–e**) Neoplastic cells are positive for HHV8 (**b**), CD138 (**c**), variable CD4 (**d**), and variable CD3 (**e**)

There were thirteen total cases of PEL submitted to the workshop, all HHV8-positive with the typical plasmablastic/immunoblastic/anaplastic morphology (Supplementary Tables 1–3, Fig. 1). Most demonstrated a terminal B/plasmablastic immunophenotype (Table 1), with three cases expressing T-cell markers (LYWS-1216, L. Mescam, Fig. 2), and two cases having both IG and TR genes clonally rearranged. Five cases were EBV-positive (Supplementary Tables 1 and 2; isolated PEL and PEL with extracavitary dissemination/tissue involvement, respectively), and similar to that reported in the literature, all were male and the majority were HIV-positive, except for one unique HIV-negative elderly male with a history of Castleman disease (LYWS-1036, L. Chen). Eight cases of EBV-negative PEL were submitted (Supplementary Tables 2 and 3; PEL with extracavitary dissemination/tissue involvement and isolated PEL, respectively), all male, two of which were HIV-positive (one of which was well controlled by the therapy), and three were post-transplant.

**Table 1:** Characteristics of the EAHP-SH workshop primary effusion lymphoma (including disseminated cases) and extracavitary primary effusion lymphoma

	Primary effusion lymphoma, EBV+ (*n* = 5)	Primary effusion lymphoma, EBV- (*n* = 8)	Extracavitary primary effusion lymphoma (*n* = 5)
M:F	5 of 5 male	8 of 8 male	5 of 5 male
Median age	35 (HIV+) 89 (HIV-)	43.5 (HIV+) 79 (HIV-) 71 (NR)	54 (HIV+) 83 (NR)
HIV	4/5 (80%)	2/5 (40%)	4/4 (100%)
Extracavitary disease	2/5 (40%)	2/8 (25%)	NA
Post-transplant	0/4 (0%)	3/8 (38%)	0/5
CD138 +	1/4 (25%)	7/7 (100%)	3/5 (60%)
CD20 +	1 (partial)/5 (20%)	0/7 (0%; one reported as weak to negative)	0/5 (0%)
IgM+	1/1 (100%)	NR	2/3 (67%)
EBV+	5/5 (100%)	0/8 (0%)	5/5 (100%)
Light chains	Kappa 1 Lambda 1 Negative 2 NR 1	Kappa or lambda 0 Negative 4 NR 4lsom	Kappa 2 Lambda 0 Negative 3
MYC rea	0/3 (0%)	0/2 (0%)	0/3 (0%)
BCL2 rea	0/3 (0%)	0/3 (0%)	0/1 (0%)
BCL6 rea	0/3 (0%)	0/3 (0%)	0/1 (0%)
Mutations	KMT2C, MAML2 (*n* = 1)	BCL6 (*n* = 1)	NR

*EBV*, Epstein-Barr virus; *M*, male; *F*, female; *HIV*, human immunodeficiency virus; *NA*, not applicable; *NR*, not reported; *rea*, rearrangement

## Extracavitary primary effusion lymphoma (ECPEL)

Although separated out here to highlight the overlap between other tissue-based HHV8-positive diseases, ECPEL is included as a subcategory of PEL in the WHO-4R, as well as the ICC and WHO-5, most commonly affecting HIV-positive males with a younger median age than PEL [10]. As noted previously, morphology and immunophenotype are similar to PEL, although expressions of CD20, CD79a, and T/NK markers are more frequent, while expressions of CD45, CD30, and EMA are less [1, 10]. The WHO-4R did not exclude EBER-negative cases; however, it did acknowledge the challenge in distinguishing ECPEL from HHV8-positive diffuse large B-cell lymphoma (HHV8+DLBCL). The WHO-5 also does not exclude EBER-negative cases from ECPEL and notes that the distinction between nodal involvement by ECPEL and HHV8+DLBCL may be difficult [4 The ICC states that HHV8+DLBCL and NOS should be favored in EBV-negative cases with cytoplasmic IgM, lambda, and/or associated with MCD. Although most cases of ECPEL are not going to create a diagnostic dilemma, rare cases are problematic.

**Fig. 3:**
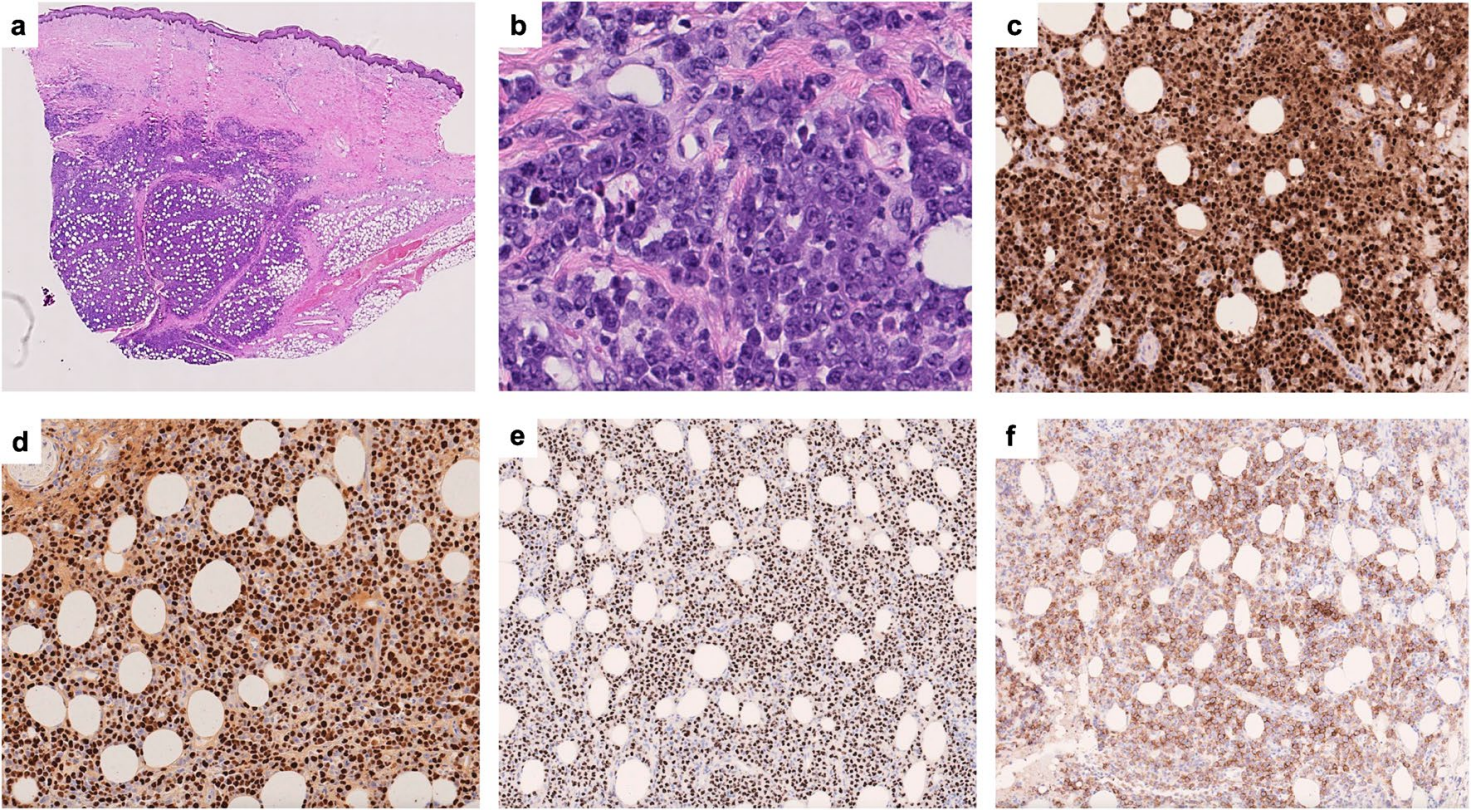
Histologic and immunophenotypic features of extracavitary primary effusion lymphoma (LYWS-1192 courtesy of A. Dashora). (**a**, **b**) Histologic sections of the subcutaneous nodule demonstrating diffusely infiltrating neoplastic lymphoid cells with immunoblastic and plasmablastic cytomorphologic features. (**c–f**) Neoplastic cells are positive for MUM1 (**c**), EBER (**d**)**,** HHV8 (**e**), and variable intensity CD138 (**f**)

Seven cases were included in the section of “ECPEL and related entities.” All patients were male, five of which were ultimately diagnosed with ECPEL (Table 1 and Supplementary Table 4). The four typical cases of ECPEL were HIV-positive and demonstrated the expected morphology and immunophenotype, although one demonstrated expression of CD4 (LYWS-1297, B. Aqil). Interestingly, LYWS-1192 (A. Dashora) presented as a subcutaneous nodule with a clinical differential diagnosis of calciphylaxis and erythema nodosum (Fig. 3). The most common sites of ECPEL presentation are lymph nodes and gastrointestinal tract, with skin presentation being uncommon (7.5% of ECPELs) [10]. However, when presenting in the skin, a subcutaneous nodule or mass has been reported most frequently [14, 15].

**Fig. 4:**
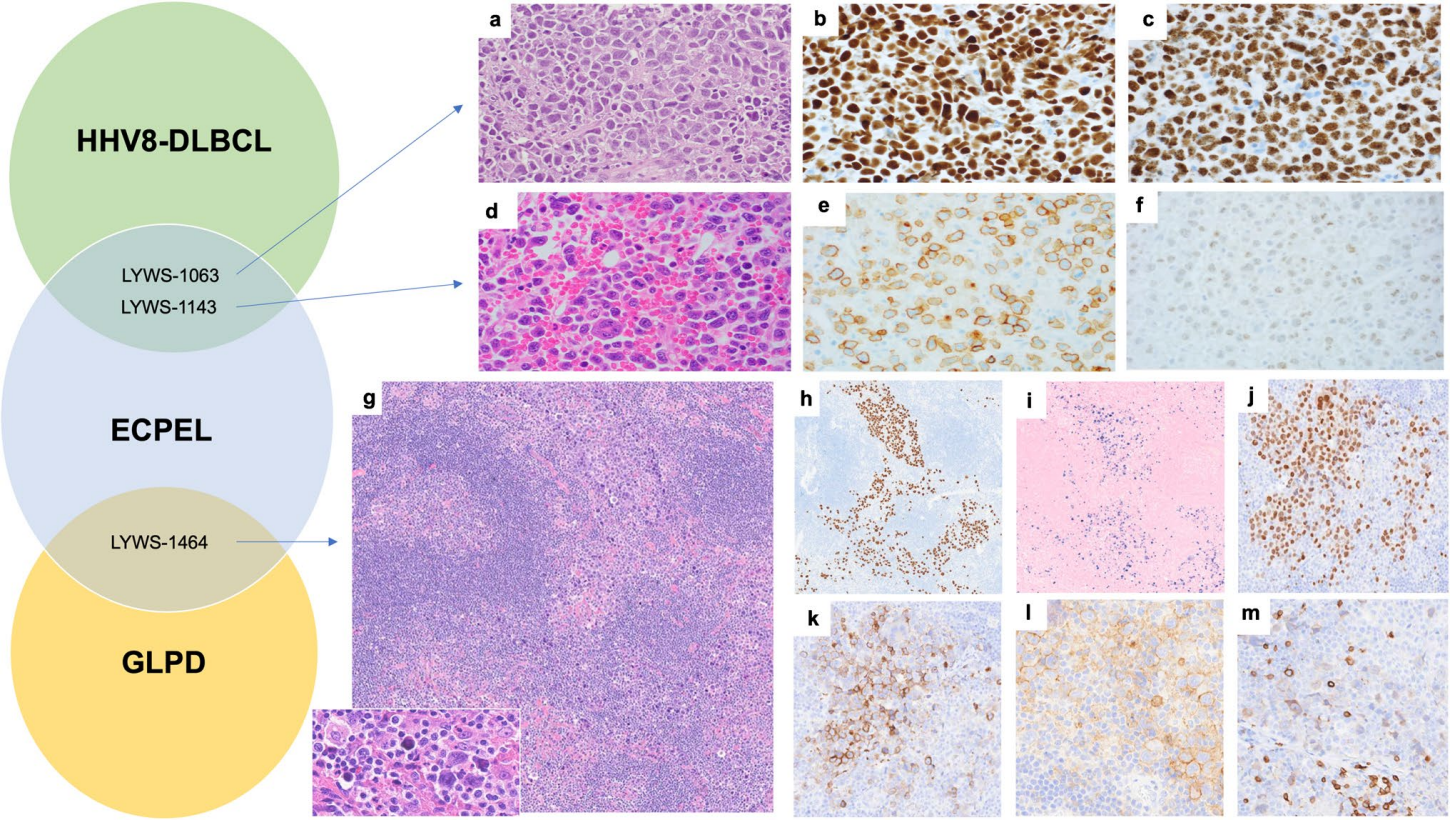
Venn diagram demonstrating overlap of HHV8+ diagnostic entities submitted to the workshop. Two challenging cases with a differential diagnosis of extracavitary primary effusion lymphoma and HHV8-positive large B-cell lymphoma, both of which were EBER negative. (**a–c)** LYWS-1063 (courtesy of K. Karube) demonstrates large, pleomorphic centroblastic/immunoblastic cells on the histologic section of the kidney (**a**), which are positive for MUM1 (**b**) and HHV8 (**c**). (**d–f**) LYWS-1143 (courtesy of L. Rimsza) demonstrates large, pleomorphic immunoblastic/plasmablastic/anaplastic cells on the histologic section of the bone marrow core biopsy (**d**), which are positive for CD138 (**e**) and weak HHV8 (**f**). A challenging case of extracavitary primary effusion lymphoma that raised consideration of germinotropic lymphoproliferative disorder (LYWS-1464 courtesy of A. Serrano). (**g**) H&E section of the inguinal lymph node. The large cell infiltrate is predominantly paracortical and sinusoidal. High power view demonstrating the plasmablastic/anaplastic cytomorphology (insert). (**h–o**) Immunophenotypic features. The neoplastic infiltrate is positive for HHV8 (**h**), EBER (**i**), MUM1 (**j**), EMA (**k**), CD38 (**l**), and subset weak CD3 (**m**)

There were three cases that created a diagnostic challenge in differentiating between ECPEL and other HHV8-positive lymphomas/lymphoproliferative disorders (Fig. 4), one of which was ultimately diagnosed as ECPEL (Supplementary Table 4). The first two cases raised the differential diagnosis of ECPEL and HHV8+DLBCL: LYWS-1063 (K. Karube, Fig. 4a–c) and LYWS-1143 (L. Rimsza, Fig. 4d–f). Both cases were positive for CD138, MUM1, and HHV8 and negative for CD20, EBER, and IgM and light chains by immunohistochemistry. LYWS-1063 was an elderly male who lived in a geographic area with a high prevalence of HHV8 (Okinowa, Japan), and LYW-1143, a 42-year-old HIV/HBV-positive male, post-liver transplant. Both patients are without a history of MCD. As noted above, although most cases of ECPEL are EBV-positive, EBV negativity is not excluded in the WHO-4R. Although the WHO-4R and ICC would regard cases with both EBV and HHV8 expression as ECPEL, there are rare reports of EBER-positive HHV8-positive lymphoid neoplasia described as DLBCL or GLPD with atypical clinical or histologic/immunophenotypic features, where some may question expanding the immunophenotypic spectrum of HHV8+DLBCL (preliminary WHO-5 online beta version notes that some HHV8+ DLBCL may be dual positive) [16, 17]. In such atypical cases, a complete study is necessary, including the mutational status of the IGHV gene. Findings typical of HHV8+DLBCL (as opposed to ECPEL) include naïve B-cell origin (somatic hypermutation negative), IgM, and lambda expression (Table 2). Both HHV8+DLBCL and ECPEL occur in the setting of HIV (or profound immunodeficiency), with MCD typical of the former. HHV8+DLBCL is generally negative for CD138, with CD20 expression in a subset of cases. Of the four straightforward cases of ECPEL in this workshop, all were EBER-positive, all were lambda light chain negative (two were kappa positive), all were CD20 negative, three were CD138 positive, and two of three cases were IgM positive (Table 1, Supplementary Table 4). A recent study of ECPEL reported ~55% with light chain restriction (kappa or lambda), ~4% with CD20, and ~77% with EBER expression [10]. Given that both cases in question were CD138 positive, and IgM and lambda negative, and neither were arising in association with MCD, the diagnosis of ECPEL was originally considered. However, because of EBER negativity (which would be unusual in the HIV+ post-transplanted case) and the lack of information regarding the mutational status of the IGHV gene, the panel could not exclude the alternative possibility of an HHV8+DLBCL with plasmablastic immunophenotype. A consensus impression was reached if we considered these cases as gray zones between the two diagnostic entities, realizing there is no WHO or ICC gray zone entity and that some would feel these two cases could be put into either ECPEL or HHV8+DLBCL without entertaining a gray zone concept.

**Table 2:** Comparison of HHV8-positive lymphomas/lymphoproliferative disorders

	Site B symptoms (Bs)	Typical morphology	B-cell stage	EBV	HHV8	Most common IP	B-cell clonality	HIV-positive patients	MCD
PEL	Body cavity effusions (can disseminate) B symptoms common	Numerous immunoblasts/plasmablasts/anaplastic cells	Terminal (SHM+)	Positive or negative Type 1 latency (LMP1-/EBNA2-)	Positive	CD20- CD138+ MUM1+ K/L-/+**	Monoclonal	Most of EBV+ cases; fewer in EBV- cases	Uncommon
ECPEL	Lymph nodes and extranodal sites B symptoms common	Effacement by diffuse sheets of immunoblasts/plasmablasts/anaplastic cells (but can be focal)	Terminal (SHM+)	Positive* Type 1 latency (LMP1-/EBNA2-)	Positive	CD20- CD138+ MUM1+ K/L+/-**	Monoclonal	Most	Uncommon
HHV8+ DLBCL	Lymph nodes, Splenomegaly common (can involve extranodal sites and blood) B symptoms common	Effacement of nodal architecture with diffuse sheets of plasmablasts/immunoblasts	Naïve, IgM+, SHM-	Negative^#^	Positive	CD20+/- CD138- MUM+ IgM+ Lambda+	Monoclonal	Most	Typical
GLPD	Localized lymph node (multifocal adenopathy uncommon but may occur) B symptoms uncommon	General retention of nodal architecture with interfollicular, polyclonal plasmacytosis. Follicles may be atrophic. Focal/non-extensive replacement of germinal centers by plasmablasts/immunoblasts/anaplastic cells. May see limited involvement of mantle zone, interfollicular region, or sinus.	Terminal (SHM+)	Positive* Type 1 latency (LMP1-/EBNA2-)	Positive	CD20- CD138- MUM+ K/L+	Polyclonal/oligoclonal^	Rare	Not reported

*Most are positive

^#^Typically considered negative, but some reports include EBER-positive cases

^Rare monoclonal cases also reported (HIV-positive patients with B symptoms and generalized adenopathy) [18]

**Although the WHO has historically considered PEL/ECPEL to be light chain absent, recent studies have shown more significant light chain expression (with a higher percentage in ECPEL than PEL) [2, 10]

*EBV*, Epstein-Barr virus; *ECPEL*, extracavitary primary effusion lymphoma; *GLPD*, germinotropic lymphoproliferative disorder; *HIV*, human immunodeficiency virus; *HHV8*, human herpes virus 8; *IP*, immunophenotype; *K/L*, kappa/lambda; *LBCL*, large B-cell lymphoma; *MCD*, multicentric Castleman disease; *PEL*, primary effusion lymphoma; *SHM*, somatic hypermutation

LYWS-1464 (A. Serrano) was an additional challenging case with a differential diagnosis between ECPEL and germinotropic lymphoproliferative disorder (GLPD) (Fig. 4g–o). The patient (83-year-old male, without effusions) was being evaluated for superficial urothelial carcinoma, and a PET avid right iliac lymph node was found, worrisome for metastatic carcinoma. Upon excision, no carcinoma was seen; however, there were foci of markedly atypical, large plasmablastic/anaplastic cells that expressed CD38, CD43, EBER, HHV8, and MUM1 and lacked CD138 and CD20 (Supplementary Table 4, note that follow-up was not available for LYWS-1464). The focal nature and lack of an identifiable clone (IGH, IGK, and IGL; not reported if microdissected) raised the question of involvement by germinotropic lymphoproliferative disorder (GLPD). However, the atypical cells were predominantly sinusoidal and paracortical, only partially involving follicles (without appearing focused in germinal centers) and without interfollicular polytypic plasmacytosis. Given the pattern and lack of light chain expression, the panel felt the findings were most in keeping with focal involvement by ECPEL; however, some authors have raised consideration that additional patterns may exist for GLPD [18–21].

The clinical setting can be helpful in differentiating GLPD from ECPEL, as GLPD typically affects elderly HIV-negative patients and ECPEL severely immunocompromised HIV-positive patients (although HIV status is not exclusive to either entity) [10, 16]. The cytomorphology and immunophenotype of GLPD overlap with ECPEL, with plasmablasts that are HHV8, EBER, and MUM1 positive and lack B-cell markers such as CD20, CD79a, and PAX5. CD138 has been reported as often positive in PEL/ECPEL and negative in GLPD; however, CD138 may not be an ideal distinguishing marker, with variable or negative ECPEL cases seen in this cohort (Table 1) and by others (~40–64% of those studied) [2, 10]. GLPD has been described as often light chain monotypic by immunohistochemistry, but polyclonal or oligoclonal by molecular studies, with the original paper evaluating clonality in two GLPD cases using microdissected foci [22], whereas ECPEL is clonal. Subsequent studies have also shown GLPD to be polyclonal or oligoclonal (although not specified as microdissected foci), with rare monoclonal cases reported (however, these are in HIV-positive patients with B symptoms and generalized adenopathy, potentially representing an alternative diagnosis of ECPEL) [18, 19]. Although, based on historical reports, the WHO-4R considers PEL/ECPEL to typically be light chain negative, more recent reports have noted light chain expression (30% in PEL and ~50–60% of ECPEL) [2, 10]. The reported findings are similar to the EA4HP workshop cohort, although interestingly, EBV-negative PEL lacked light chain expression in those tested (Table 1). Alternatively, GLPD commonly expresses light chain (thirteen of fifteen cases tested, with kappa versus lambda essentially equivalent) [19]. Given the overlap in cytomorphology and immunophenotype, the morphologic pattern is often the most useful diagnostic feature in GLPD: general retention of nodal architecture and involvement of germinal centers by medium to large plasmablastic cells [1]. When alternative patterns have been reported, such as sinusoidal, mantle, and interfollicular involvement, this is usually in addition to germinal center involvement [8, 18–20], although not always [21]. A recent review [19] found that interfollicular polytypic plasmacytosis was a common feature in GLPD (~37%), which has also been reported as a prominent finding by others [18, 22].

There are a few intriguing reported patients, somewhat like LYWS-1464 (A. Serrano). One is an elderly HIV-negative patient with incidental lymph node findings of sinus involvement by HHV8/EBV/MUM1/Kappa/IgM+ plasmablastic cells that were clonal on microdissection [23]. The patient was treated with chemotherapy, but 18 months later developed PEL. Of interest, no additional therapy was given after thoracentesis, and the patient was alive without recurrence (8 months of follow-up). An additional reported patient (elderly HIV-negative) where nodal involvement was limited (only scattered large atypical HHV8/EBV-positive cells, IGH PCR was polyclonal) relapsed 55 months post-therapy [24]. Both reported cases demonstrated that incidental findings and limited involvement may show progression after therapy. Given the potential overlap of GLPD with ECPEL in some cases, close clinical follow-up with clinical-pathologic discussion on the best approach (and potential biopsy of multiple sites) is prudent. Continued reporting of these unusual and challenging cases (ideally with therapy and follow-up) will be of help in further refining the diagnostic criteria.

These last three cases highlighted the challenges in specific diagnosis and the spectrum of findings that can be seen in HHV8-positive lymphoid proliferations/neoplasms (Table 2) [1, 2, 16–18, 23, 25].

## HHV8-negative lymphomas, effusion-based/presenting as an effusion

This was a challenging area for the panel, given it is a newly proposed or provisional entity that is uncommon, with diagnostic criteria that are continuing to evolve. Cases under the umbrella of KSHV/HHV8-negative effusion lymphomas have been reported under a variety of names (such as HHV8-negative PEL, PEL-like lymphoma, primary HHV8-negative effusion-based lymphoma, type II PEL) and include a variety of clinical settings and immunophenotypes, although fluid retention has been considered a potential etiology in a number of patients [26–30]. These effusion-based lymphomas have more commonly been reported in Asian countries (particularly Japan), in HIV-negative elderly patients and in fluid overload conditions with pleural effusion being the most common site. HHV8-negative effusion-based lymphomas were not a specific entity in the WHO-4R; however, it was mentioned in the PEL chapter to avoid misclassification of KSHV/HHV8-negative effusion-based lymphomas as PEL, given the apparent better prognosis and different clinical setting [1, 28, 29]. Unlike most reports on PEL, at least subsets of HHV8-negative effusion-based lymphomas have survivals similar (or superior) to nodal-based DLBCL (Supplementary Table 5). Unlike nodal-based DLBCL (and PEL), remarkably, some cases of HHV8-negative effusion-based lymphomas have a complete response to drainage alone [29]. Currently, the WHO-5 separated these cases out as a specific diagnostic entity, “fluid overload-associated large B-cell lymphoma (FO-LBCL),” and the ICC recognizes “HHV8 and EBV negative primary effusion based lymphoma (HHV8negEBVneg-PEBL)” as a provisional entity [4, 5]. Features in both classifications include morphology that can be similar to PEL (immunoblastic/centroblastic/anaplastic), elderly HIV-negative patients with medical conditions that lead to fluid overload, without adenopathy or mass lesions. However, there are differing opinions on immunophenotype (IP), and neither classification addresses the post-transplant setting. The ICC requires EBV negativity and notes that most of these lymphomas express at least one B-cell marker to avoid the inclusion of plasmablastic neoplasms [5]. The WHO-5 manuscript notes a mature B-cell rather than a plasmablastic IP and that EBV is positive in 13–30% of cases [4].

There were fifteen cases submitted that were KSHV/HHV8-negative lymphomas presenting as an effusion that raised the question of FO-LBCL/HHV8negEBVneg-PEBL; however, given the new and evolving nature of the category, this was separated out into three categories by the panel: EBV-negative cases with a B-cell IP (seven cases), EBV-positive cases with a B-cell IP (three cases), and cases with a PB IP and morphology (five cases; four of which were EBV-positive).

**Fig. 5:**
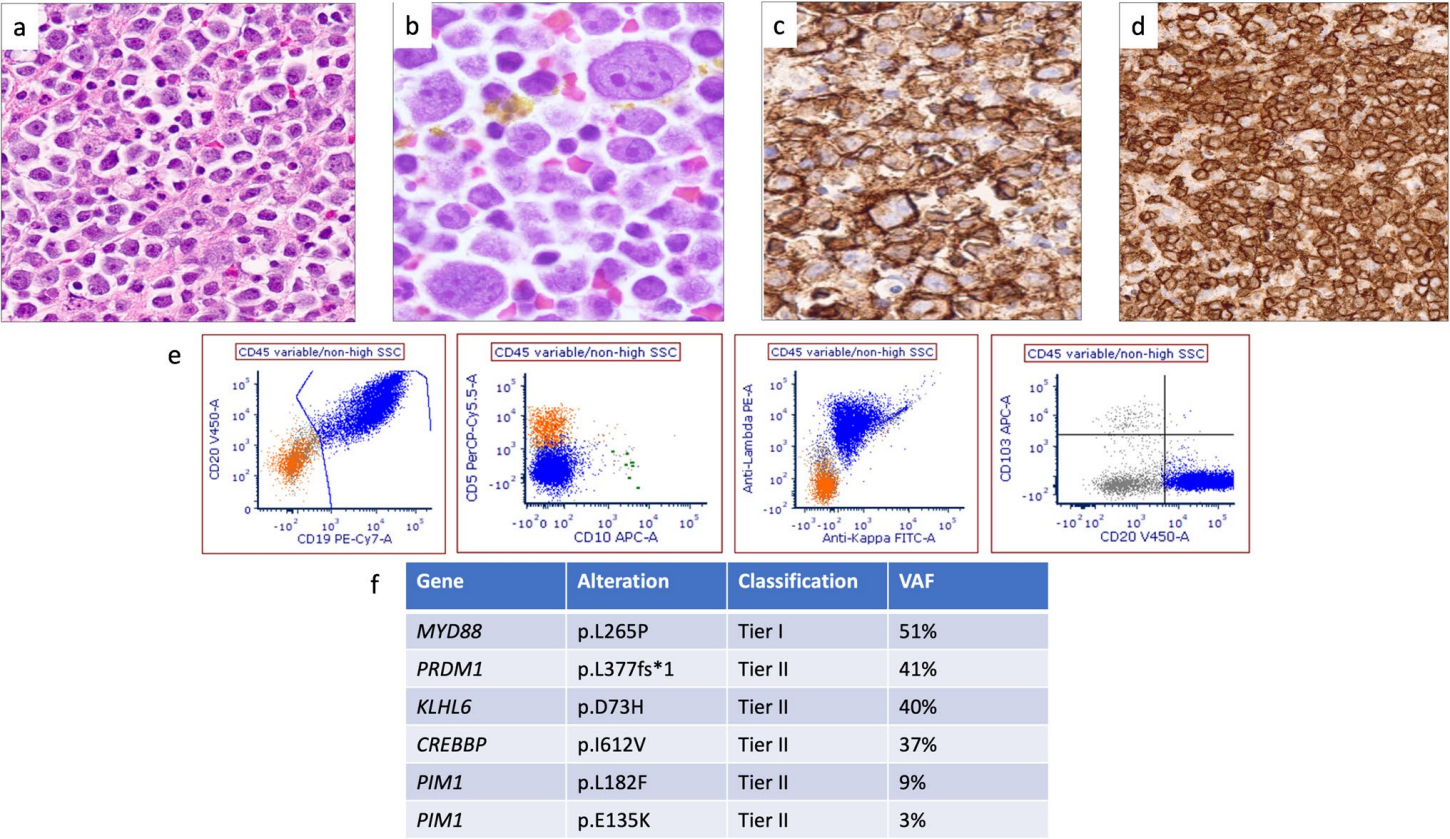
HHV8-negative and EBER-negative effusion-based lymphoma/fluid overload large B-cell lymphoma (LYWS-1065 courtesy of N. Aggarwal and A. Davis). (**a**, **b**) Histologic sections of the thoracentesis cell block demonstrated large immunoblastic and plasmacytoid/plasmablastic cells. (**c**, **d**) The neoplastic population is positive for CD20 (**c**) and CD138 (**d**). (**e**) Flow cytometry demonstrates expression of CD19 and CD20 and lambda light chain without CD5, CD10, or CD103. (**f**) Next-generation sequencing demonstrates mutations that have been reported in effusion-based lymphoma (including MYD88 and PIM1)

The seven EBV-negative, CD20-positive cases were all in elderly patients (74–90 years old), five females and two males, with medical conditions that would predispose to fluid overload (Supplementary Table 6). Clinical and pathologic findings were similar to that reported in the literature (Table 3). Interestingly, two cases were also CD138 positive (LYWS-1065 A. Davis, Fig. 5, and LYWS-1243 A. Volaric), both of which expressed CD20 and PAX5. The genomic landscape has been reported to be complex and similar to conventional DLBCL, with frequent mutations (commonly *HIST1H1E* and *MYD88*), copy number alterations, and translocations (most frequently *MYC*, *BCL2*, *BCL6*) [31]. Of the two cases with reported NGS, both had multiple mutations, including one with *MYD88*^*L265P*^, and three of four cases demonstrated *BCL6* rearrangement (Table 3; Supplementary Table 6).

**Table 3:** Clinical and pathologic findings of the EAHP-SH workshop HHV8-negative effusion-based lymphoma cases and comparison to the literature

	EAHP-SH WS 2022 EBV-negative B-cell phenotype, effusion only (*n* = 7)	EAHP-SH WS 2022 EBV-positive B-cell phenotype, effusion only (*n* = 2)	EAHP-SH WS 2022 Plasmacytic-Plasmablastic phenotype (*n* = 5)	Gisriel 2022 [30] (study cohort and literature, *n* = 202)*	Kubota 2018 [32] (study cohort and literature, *n* = 67)*
M:F	2:5	2 of 2 male	4:1	113:89	42:25
Median age	80	43	59	79	80
HIV	0/3 (0%)	NR	1/1 (100%)	3/125 (2%)	3/61 (4.9%)
HCV	NR	NR	Pos 1/1	Pos 11/129	9/54 (16.7%)
Pleural effusion	6/7 (86%)	1/2 (50%)	1/5 (20%)	153/202 76%)	47/67 (70.1%)
Pericardial effusion	4/7 (57%)	1/2 (50%)	1/5 (20%)	77/202(38%)	16/67 (24%)
Peritoneal effusion	0/7 (0%)	0/2 (0%)	3/5 (60%)	24/202 (12%)	20/67 (30%)
Clinical setting of fluid overload	7/7 (100%)	1/2 (50%)	5/5 (100%)	89/158 (56%)	
EBV+	0/7 (0%)	2/2 (100%)	4/5 (80%)	16/171 (9%)*	15/65 (23%)*
Immune def/supp		2/2 (100%)	3/4 (75%)	NR	11/15 (73%)
CD20+	7/7 (100%)	2/2 (100%)	0/5 (0%)	182/190 (96%)	52/67 (29.9%)
CD10+	1/7 (14%)	0/1 (0%)	NR	24/152 (16%)	10/67 (15%)
MUM1+	6/6 (100%)	1/2(50%)	4/5 (80%)	84/112 (75%)	14/67 (21%)
CD138+	2/5 (40%)	NR	4/5 (80%)	6/97 (6%)	7/67 (10%)
MYC rea	0/5 (0%)	0/1 (0%)	3/3 (100%)	20/107 (19%)	4/27 (15%)
BCL2 rea	0/4 (0%)	0/1 (0%)	0/2 (0%)	6/54 (11%)	NR
BCL6 rea	3/4 (75%)	0/1 (0%)	0/2 (0%)	16/54 (29%)	NR
Double or triple hit	0/4 (0%)	0/1 (0%)	0/3 (0%)	6/100 (6%)	NR
Mutations	CCND3, CD58, CREBBP, IGLL5, KLHL6, MAP2K1, MYD88, NFKBIA, NOTCH2, PIK3CA, PIM1, PRDM1, RB1, TNFAIP3, ZNF292 (n=2)	No tier 1–2 reported	POT1, TP53, XPO1 (*n* = 1)	NR	NR

*M*, male; *F*, female; *HCV*, hepatitis C virus; *HIV*, human immunodeficiency virus; *EBV*, Epstein-Barr virus; *rea*, rearrangement; *NR*, not reported; *immune def/sup*, immune deficiency/suppression

*The case reported by Ashihara E et al. (International Journal of Hematology.74(2001):327-332) should be currently re-classified as FA-DLBCL since it developed in an abdominal cystic cavity associated with a ventriculoperitoneal shunt tube

**Fig. 6:**
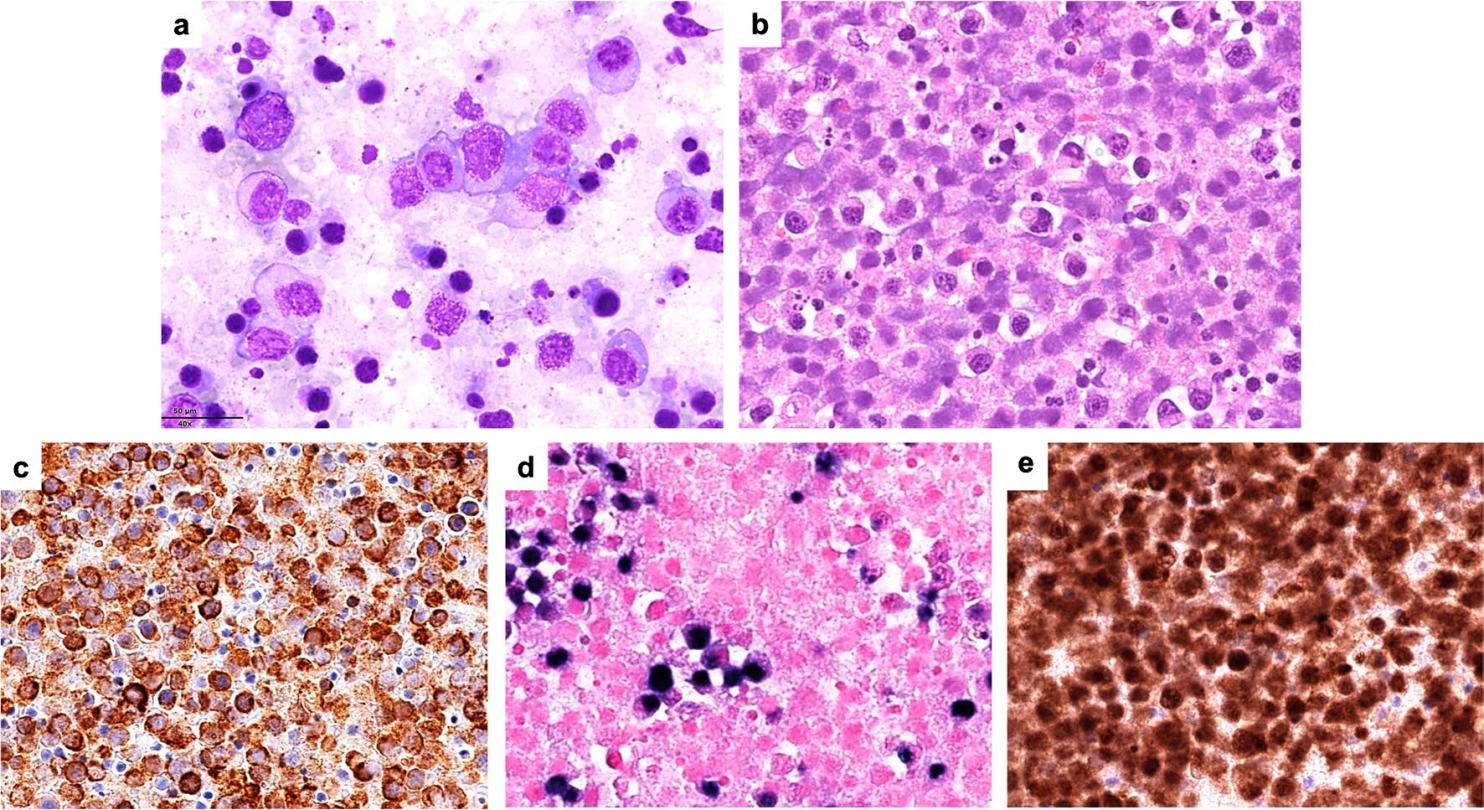
HHV8-negative and EBV-positive large B-cell lymphoma presenting as an effusion in a CML patient on Dasatinib (LYWS-1161 courtesy of L. Veloza). (**a**, **b**) Cytospin and histologic section from cell block of pericardial fluid demonstrating large immunoblastic and plasmacytoid/plasmablastic cells. (**c**–**e**) The neoplastic cells are positive for CD79a (**c**), EBER (**d**), and EBNA2 (**e**)

There were two cases of EBV-positive effusion-based/effusion-only large B-cell lymphomas (CD20 positive) without reported mass/adenopathy, both males in their 40s in a somewhat different clinical setting than EBV-negative cases (Table 3; Supplementary Table 7). These cases provided another challenge in best classification; however, the panel felt that EBV-positive diffuse large B-cell lymphoma or polymorphic EBV-positive lymphoproliferative disorder (LPD) (presenting as an effusion) was the best classification since it could be argued whether these were in the setting of immunodeficiency given an associated neoplasm (CML post dasatinib, LYWS-1161 L. Veloza, Fig. 6) and immunotherapy (LYWS-1088 M. Chiselite). Dasatinib has been associated with pleural effusions [33], with a handful of case reports describing lymphomatous effusions, all with CD20 expression but the variable expression of EBV [34–37]. The other EBV-positive cases reported in the literature with detailed clinical history occurred mostly in elderly immunodeficient (e.g., HIV, post-transplant, common variable immunodeficiency, idiopathic CD4+ T lymphocytopenia) or immunosuppressed patients (e.g., prednisone, cyclosporine) [29, 32]. Further reporting and assessment of these cases will help to refine diagnostic criteria of lymphomas in this unique setting. An additional case favored to represent EBV-positive DLBCL by the panel, although different from the prior two cases discussed with effusions only, is LYS-1075 (K. Rech, Supplementary Table 7). This case raised a differential that included FA-DLBCL, given the neoplastic cells were focally within fibrin upon decortication (thoracentesis was unsuccessful). However, the blood was EBV PCR positive, the PET scan was avid at other sites (although low avidity), and disseminated EBV-positive DLBCL could not be excluded.

**Fig. 7:**
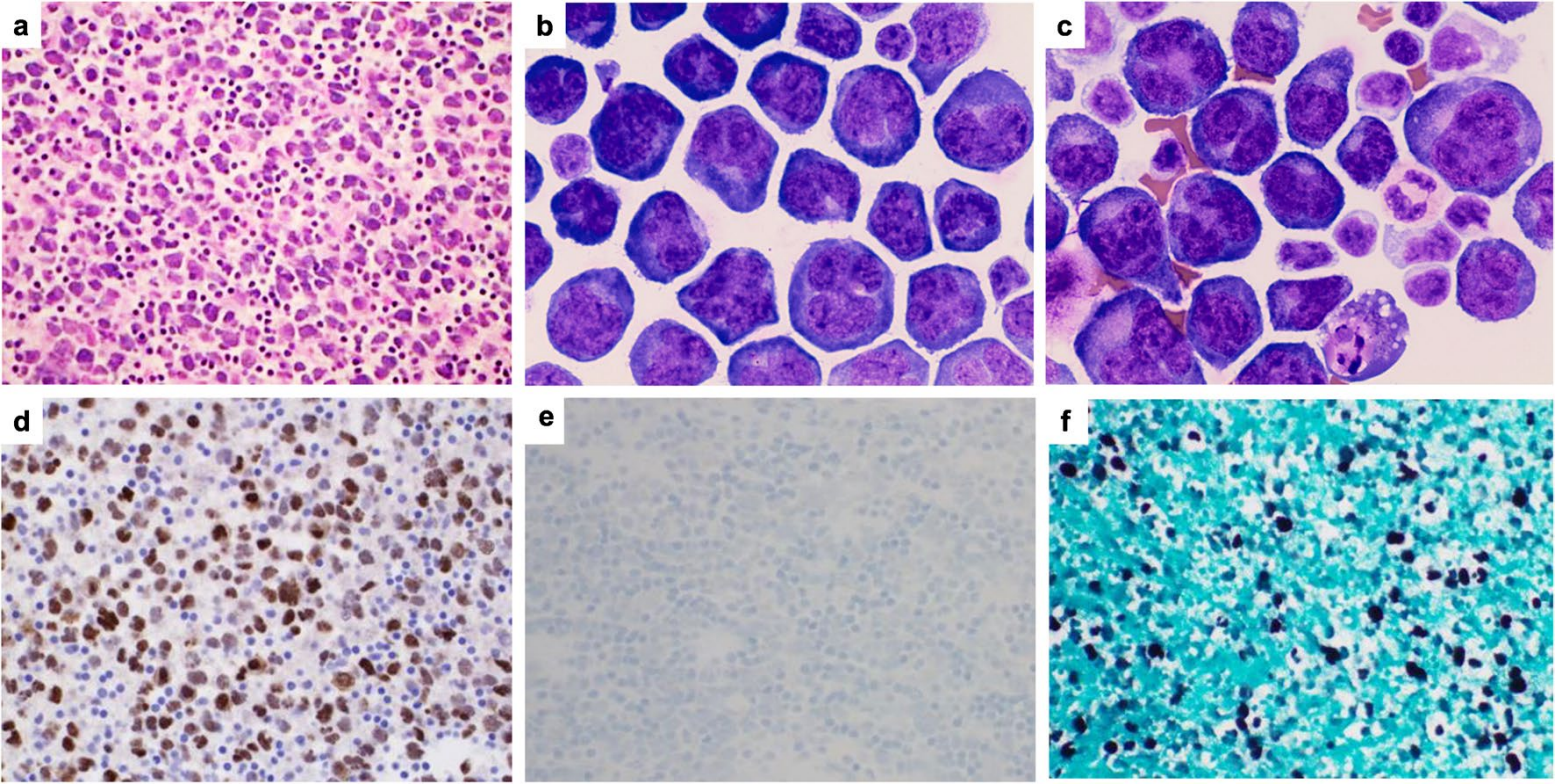
HHV8-negative and EBV-positive plasmablastic lymphoma, presenting as an effusion, post-transplant (LYWS-1382 courtesy of B. Mai and W. Wang). (**a–c**) Cell block and cytospins of the ascitic fluid demonstrates large immunoblastic/plasmablastic/anaplastic cells. (**d–f**) The neoplastic cells are positive for MUM1 (**d**), negative for HHV8 (**e**), and positive for EBER (**f**)

Five cases presenting as an effusion with a plasmacytic/plasmablastic immunophenotype (PBIP) and morphology were submitted, three with effusions only, two with tissue involvement, and one overt (Table 3; Supplementary Table 8). One patient was HIV+, and two were liver or kidney transplanted. Four out of 5 were EBER-positive. The three cases that were effusion-based only were a classification dilemma, all of which were EBV-positive, one of which was post-transplant (LYWS-1382, W. Wang, Fig. 7). One patient was particularly unusual (LYWS-1055 D. Jevremovic), with the neoplasm displaying areas with typical plasma cell morphology and at least 3-year survival (typical median survival 6-11 months for plasmablastic lymphoma, PBL) [1]. After much discussion, the panel felt these cases best fit with PBL given the morphology, immunophenotype (including EBER expression in four of the five cases), and *MYC* rearrangement.

HHV8-negative effusion-based lymphoma classification schemes have been proposed [27, 38], with a recent multi-institutional case series evaluating a large cohort that, unlike prior studies, specifically excluded patients with PBIP, history of low-grade B-cell lymphoma, and solid organ transplant [30]. Interestingly, CD20 expression has been reported as a significant, independent favorable prognostic indicator, although it is unclear if the lack of CD20 is additionally associated with PBIP [32]. Although the historic literature has reported a good prognosis [28], other studies (particularly in non-Japanese cohorts) did not demonstrate superior outcomes (Supplementary Table 5); however, it is difficult to tease out lymphoma-specific death in some studies, given the confounding comorbidities of these typically elderly patients. It will be of interest for future studies to consider evaluating inclusion/exclusion criteria, such as *MYC* rearrangement (including double-hit status), PBIP, EBV positivity, history of lymphoma, organ transplantation, and medical predisposition for fluid overload, to further support/refine or refute this new diagnostic entity.

## Other lymphomas presenting as an effusion

Four cases (3 ALCL, 1 DLBCL) were submitted that were not typical cavity-based lymphomas; however, they presented as an effusion (Supplementary Table 9). These cases exemplify the need to evaluate for and exclude tissue-based lymphoma with secondary cavity involvement.

## Diffuse large B-cell lymphoma associated with chronic inflammation (CI-DLBCL)

CI-DLBCL is an EBV-associated large B-cell lymphoma arising in the setting of persistent (>10 years) chronic suppurative inflammation involving confined natural or acquired body spaces [1, 4, 5]. The prototype is pyothorax-associated lymphoma (PAL), mostly reported in Japan that develops in the pleural cavity of patients with pyothorax secondary to artificial pneumothorax as therapy for tuberculosis [39]. Other cases have been reported within bone, joint, and skin in the settings of chronic osteomyelitis, metallic implants, surgical mesh, and chronic stasis ulcer [40–42]. CI-DLBCL affects adult patients, mostly male (M:F = 12:1; median age = 70 years old, range 50–80) [39, 43, 44]. It usually presents as a tumor mass composed of large cells associated with necrosis and fibrosis of the affected tissue. Tumor cells show centroblastic or immunoblastic morphology and an activated B-cell phenotype (CD20+, CD79a+, CD10-, BCL-6-, MUM1+, CD30 +/-). However, some cases may show plasmacytic differentiation with weak or negative CD20 and positivity for CD138, as well as aberrant expression of T-cell antigens (CD2, CD3, CD4, CD7) [3, 39, 43, 44]. Type III EBV latency profile (EBER+/LMP-1+/EBNA-2+) is characteristic [42, 44]. CI-DLBCL demonstrates a complex karyotype, common *MYC* amplification, and frequent *TP53* mutations [41, 45–47]. Clinical outcome is mostly available for PAL cases with a dismal prognosis (median survival time of 5 months) [39, 43].

There were no cases submitted that the panel felt fit the criteria for inclusion as CI-DLBCL.

## Fibrin-associated DLBCL (FA-DLBCL)

FA-DLBCL, considered a subtype of CI-DLBCL in the WHO-4R [1] and by the ICC [5], has been recognized as a distinct entity in the WHO-5 [4]. It consists of non-mass-forming aggregates of large atypical lymphoid cells in a background of debris or fibrin, usually encountered incidentally in confined, natural, or acquired spaces such as cardiac myxomas, chronic hematomas, thrombi, cardiovascular prosthetic devices [48–50], cysts and pseudocyst cavities (including those forming around breast implants) [48, 51, 52], and pacemakers [53, 54]. Tumor cells may focally infiltrate the adjacent stroma (myxomatous or fibrotic) but not the pre-existing normal tissues. Median age is 56 years (range 25–96) with male predominance (M:F ratio 2:1), although those associated with breast implants were all female with a median age of 65 years (range 47–71). Time from placement of devices to lymphoma diagnosis is variable (range 4–26 years for breast implants [55]; range 1–20 years for other devices) [49]. The majority of cases are associated with excellent outcomes regardless of therapy (surgery alone or chemotherapy), although a few patients with primary cardiac or vascular disease experience recurrent or persistent disease [48]. Neoplastic cells typically express at least two B-cell markers (CD20, CD79a, PAX5) and a non-GCB phenotype (CD10−, BCL6-, MUM1+). CD30 may be expressed, with plasmacytic immunophenotypic features (CD20-, PAX5-, CD38+, and intracytoplasmic monotypic light chain) rarely reported [56]. The tumor usually demonstrates latency type III EBV infection, with rare cases being EBV-negative [57–59]. As with CI-DLBCL, local immunodeficiency induced by chronic inflammation is thought to favor immune evasion of EBV-transformed B-cells. Expression of high levels of IL-10, TGF-β1, IL-35, and PD-L1 by EBV latency type III-immortalized B-cells support this hypothesis [40, 41, 60, 61]. No *MYC* rearrangements (or >2 extra copies) have been reported [48].

**Fig. 8:**
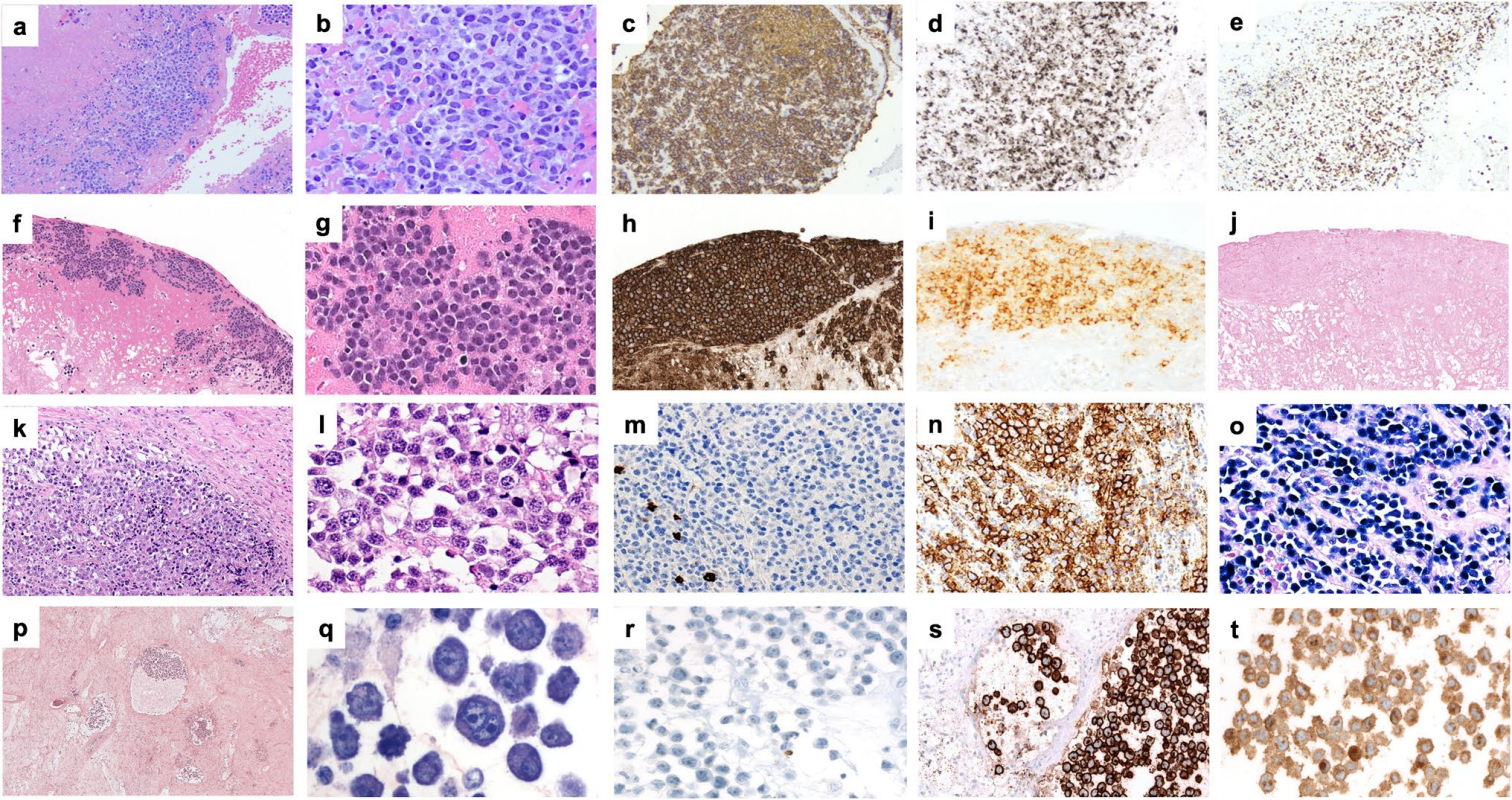
Histologic and immunophenotypic features of FA-DLBCL involving different anatomical sites. (**a**–**e**) Large non-GCB-type B-cells in a left femoral artery thrombi (LYWS-1166 courtesy of R Morse), (**a**, **b**) H&E, (**c**) CD20, (**d)** MUM1, and (**e)** EBER; (**f–j**) Large GCB-type B-cells infiltrating a cardiac myxoma (LWYS-1032, courtesy of AM Perry), (**f**, **g**) H&E, (**h)** CD20, (**i)** CD10, and (**j)** EBER; (**k**–**o**) Plasmablastic cells infiltrating the fibrous psudocapsule of a pacemaker pocket (LYWS-1307, courtesy of J. Goodlad and G. Horne), (**k**, **l**) H&E, (**m)** CD20, (**n)** CD138, and (**o)** EBER; (**p–t**) Plasmablastic cells within pseudocysts of a post-therapy GIST (LYWS-1122 courtesy of F. Fend) (**p**) H&E, (**q**) Giemsa, (**r**) CD20, (**s)** CD138, and (**t)** lambda

Twenty cases of FA-DLBCL were submitted to the workshop (Table 4 and Supplementary Table 10): 10 cases involving the cardiovascular system and 10 found within cysts/pseudocysts. Ten patients with grafts/implants included valvular (1 case), vascular (3 cases), breast (5 cases), and pacemaker (1 case) with a median time from surgery to lymphoma of 12.5 years. Four cases were EBV-negative: three cardiac myxomas and one gastrointestinal stromal tumor (GIST). PD-L1 was tested in 4 cases and was negative in the EBV-negative case. The majority of the submitted cases expressed B-cell lineage markers and a non-germinal center B (GCB)-type phenotype (Fig. 8a, e); however, 2 cases were GCB-type (LWYS-1032, A.M. Perry, Fig. 8f–j; LWYS-1384 a. Padrão), and 2 showed plasmablastic morphology and phenotype (CD20-, CD138+) (LWYS-1307, J. Goodlad and G. Horne and LWYS-1122, F. Fend, Fig. 8k–t). LYWS-1122 was a challenging case, where the differential diagnosis included plasmablastic lymphoma (PBL) and FA-DLBCL arising in a post-therapy (avapritinib) pseudocyst of a *PDGFRA*^D842V^-mutated gastrointestinal stromal tumor (GIST). Although the lymphoma cells showed a plasmablastic phenotype and *MYC* rearrangement, against PBL were negativity for EBV, expression of IgM, incidental nature of the finding, non-infiltrative pattern of growth, and non-aggressive clinical behavior of the disease with complete remission after surgical resection of the post-therapy GIST (albeit only one year of follow-up).

**Table 4:** Characteristics of the EAHP-SH workshop fibrin-associated diffuse large B-cell lymphoma cases

Site	Cardiac myxoma (*n* = 3)	Thrombus (*n* = 7) - valves (*n* = 2) - vascular (*n* = 5)	Cyst/pseudocyst (*n* = 10) - Adrenal (*n* = 2) - Hepatic (*n* = 1) - GIST (*n* = 1) - Pacemaker pocket (*n* = 1) - Breast implant (*n* = 5)
M:F	1:2	6:1	3:5 (2 NR)
Median age (range)	60 (50–76)	72 (23–76)	61 (45–72)
Graft/foreign body	-	4/7 (57%)	6/9 (67%)
Median time from implantation (years) (range)	-	14.5 (0.6-23)	12.5 (1.6-26)
HIV	NR	NR	1 (HIV+) 9 (NR)
EBV+	0/3 (0%)	7/7 (100%)	9/10 (90%)
CD20 +	3/3 (100%)	6/7 (1 partial) (86%)	8/10 (1 partial) (80%)
CD10+	2/3 (67%)	0/7 (0%)	0/5 (0%)
MUM1+	1/3 (33%)	7/7 (100%)	8/8 (100%)
CD138+	NR	0/2 (0%)	4/4 (1 partial) (100%)
CD30+	0/2 (0%)	4 (3 partial)/6 (67%)	7/10 (1 partial) (70%)
PD-L1+	0/1 (0%)	1/1 (100%)	2/2 (100%)
MYC rea	0/3 (0%)	0/3 (0%)	1/3 (33%)
BCL2 rea	0/3 (0%)	0/3 (0%)	0/3 (0%)
BCL6 rea	1/3 (33%)	0/2 (0%)	0/3 (0%)
Mutations	BCL11A, CARD11, CD58, CD79B, CREBBP, ETV6, HIST1H1E, HIST1H2BD, HIST1H1D; HLA-B, IKZF3, NOTCH1, PAX5, PIM1 (n=2)		BTG1, CXCR4, KMT2D, MEF2B (n=1)

*M*, male; *F*, female; *HIV*, human immunodeficiency virus; *EBV*, Epstein-Barr virus; *NR*, not reported; *rea*, rearrangement

**Fig. 9:**
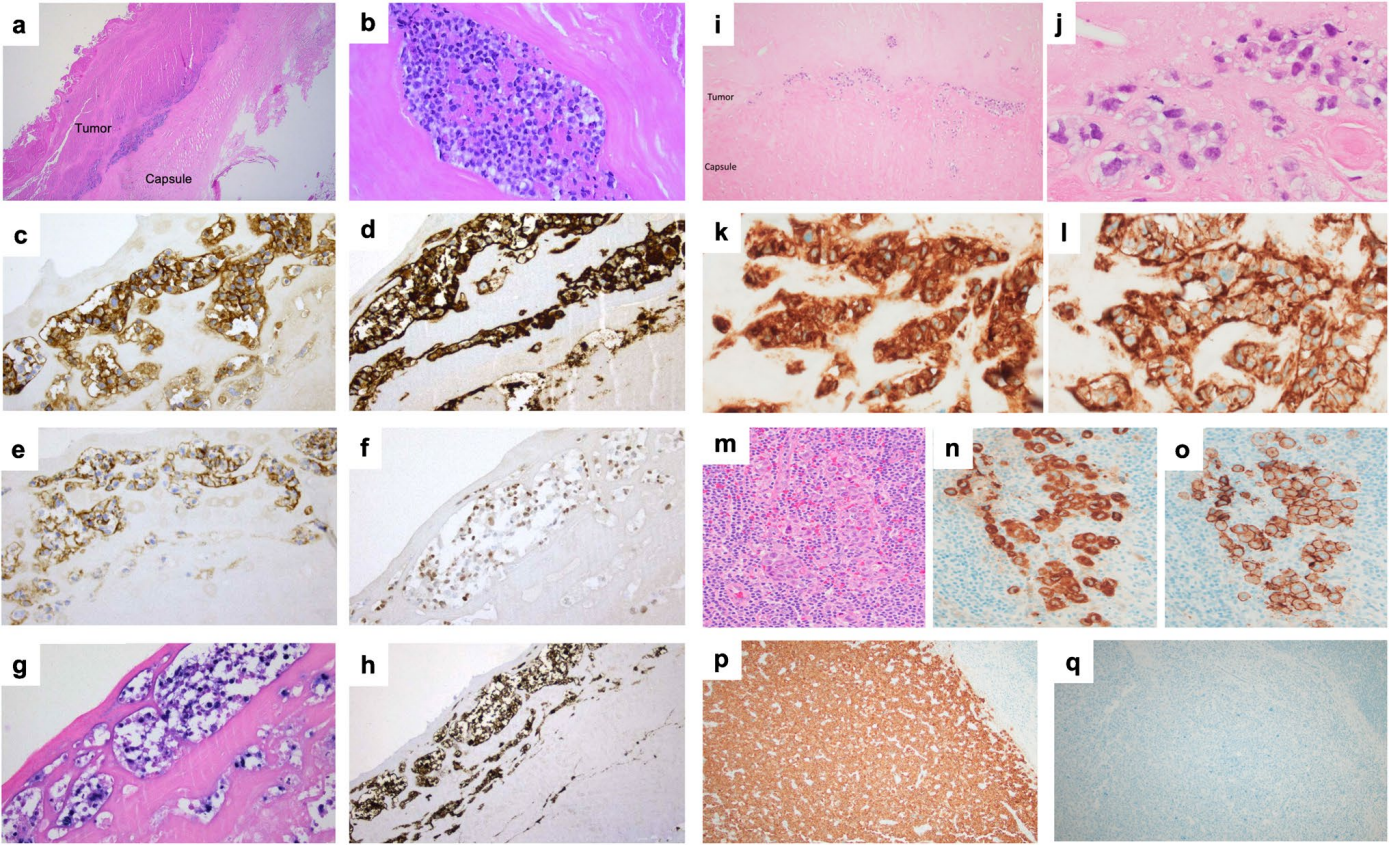
Large cell lymphomas associated with breast implants. (**a**–**h**) FA-DLBCL represented by clusters of EBV-infected large B-cells within fibrin infiltrating the capsule (LWYS-1097, courtesy of T. Tousseyn) (**a**, **b)** H&E, (**c**) CD30, (**d**) CD20, (**e**) CD19, (**f**) PAX5, (**g**) EBER, and (**h)** LMP1. (**i–q**) BIA-ALCL composed of aggregates of large anaplastic cells associated with fibrinous material infiltrating the capsule (LYWS-1124 courtesy of A.L. Feldman). (**i–l**) H&E and the axillary lymph node (**m**–**q**) H&E. Tumor cells show strong expression of both CD30 and CA9 in the capsule (**k**, **l**) and in the small lymph node tumor aggregates (**n**, **o**) but were CD30-positive and CA9-negative (**p**, **q**) in the diffuse areas in the lymph node

Only one of the FA-DLBCL occurred in an HIV-positive patient (under control with therapy) in association with breast implants (LYWS-1097, T. Tousseyn, Fig. 9a–h).

Therapy and follow-up were not available for many of the cases; however, most of the patients with FA-DLBCL associated with thrombi/fibrin in vessels, vascular grafts, or valve replacements did not fare well (seven patients with follow-up). Three patients died secondary to complications of surgery, additional thrombotic events, or secondary CNS involvement (mitral valve presumed origin). One patient was treated with rituximab, cyclophosphamide, vincristine, and prednisone (R-CVP), developed a lung mass, and subsequently died. The other three were treated with rituximab, cyclophosphamide, doxorubicin, vincristine, and prednisone (R-CHOP) (two because of progressive disease, one with presumed CNS involvement), with one reporting no evidence of disease at 3 months and one developing an EBV-negative LBCL 5 years later. In contrast, two of the FA-DLBCL cases associated with myxoma (24 years and 11 months follow-up) and three occurring within cystic/pseudocystic spaces (6 years, 1 year, and 6 years follow-up) did not recur after surgery alone. This observation is intriguing and may indicate that the prognosis of FA-DLBCL may be influenced by the site of origin, with the vascular compartment potentially having the highest risk for systemic involvement and complications. A similar hypothesis was previously made by Boyer et al., who noticed that out of twelve cases, the three with recurrent or persistent disease were associated with vascular thrombi or mitral valve (initial site atrial myxoma for the latter) [48]. In contrast, Zanelli et al. reported an FA-DLBCL in a cerebral artery aneurysm with a benign clinical course after surgery [49]. Additional reports are needed to draw definitive conclusions.

## Breast implant-associated anaplastic large cell lymphoma (BIA-ALCL)

BIA-ALCL, previously considered a provisional entity within the group of the anaplastic large cell lymphomas [1], has become a definitive entity in both WHO-5 and ICC [4, 5]. It occurs in women (median 53 years, range 24–90) implanted with breast prosthesis either for esthetic or reconstruction for breast cancer [62]. Similar to the FA-DLBCL, it usually manifests as a late-onset peri-implant seroma (median 8 years) in which pleomorphic and anaplastic large cells grow within a necrotic-fibrinous background. However, about 10% of patients present with a tumor mass infiltrating the peri-implant tissues and/or with regional lymphadenopathy [62]. Indeed, BIA-ALCL is currently staged according to the TNM system as T1: tumor cells in seroma and/or on the capsular luminal surface; T2: early capsule infiltration; T3: massive capsule infiltration; and T4: infiltration beyond the capsule [63]. The immunophenotype of tumor cells overlaps with that of other types of ALCL with strong CD30 expression, positivity for cytotoxic markers, frequent CD4 expression, and negativity or focal positivity for T-cell-associated antigens CD3, CD5, and CD7. EBV and ALK are consistently negative. Similar to other ALCLs, monoclonal rearrangement of the TR genes is present in most cases, and although T-cell receptor signaling is downregulated, the STAT3 pathway is upregulated with phosphorylation of the STAT3 protein [64–66]. In contrast to other ALCLs, a hypoxia gene signature with higher CA9 expression by the tumor cells has been observed in BIA-ALCL [67]. Chronic inflammation is also thought to play a role in BIA-ALCL development. Somatic mutations in genes involved in the JAK/STAT3 pathway (i.e., *STAT3, STAT5B*, *JAK1*, *JAK2*, *SOCS1*, and *SOCS3*), genes controlling the epigenetic machinery (i.e., *KMT2C*, *KMT2D*, *CHD2*, *CREBBP*, and *DNMT3A*), and *TP53* have been detected in the majority of cases [65, 68–72]. Cytogenetic abnormalities include loss of chromosome 20 [73] and amplification of PD-L1 [74]. Localized BIA-ALCL has an excellent prognosis with surgery alone, whereas immuno/chemotherapy is needed in advanced diseases [63, 75, 76].

The workshop received 9 cases of BIA-ALCL that developed after a median time from implantation of 10 years (8–29) (Table 5, Supplementary Table 11). All reported patients were female with a median age of 49 years (range 32–66). In 4 cases (44%), the disease was confined to the seroma or capsule, whereas in 5 cases (56%), it formed a mass and/or was disseminated to lymph nodes. Tumor cells showed the typical morphology and phenotype (CD30+, ALK-, PAX5-, EBER-) with all but one being CD3-negative and all but one expressing CD4 (at least focally). Expression of pSTAT3 was documented in one case, and two cases demonstrated CA9 expression. Interestingly in one case, CA9 staining was strong in the capsule sample and in focal lymph node tumor aggregates, whereas the diffuse tumor cell infiltrate in the lymph node was negative (LYWS-1124 A. Feldman, Fig. 9i–q). TR gene rearrangement was monoclonal in all 5 cases tested. No rearrangements of *IRF4/DUSP22* or *TP63* were found by FISH in 4 and 1 cases, respectively, supporting previous reports [65, 77, 78]. Targeted mutational analysis (LYWS-1428 Dr. Auclair) revealed mutations in *STAT3*, *KMT2A*, *EPHA3*, and *MALT1*. A *NOTCH2* variant with unknown significance was reported in another case (LYWS-1098 Y. Bühler).

**Table 5:** Characteristics of the EAHP-SH workshop breast implant-associated anaplastic large cell lymphoma cases

Features	BIA-ALCL (*n* = 9)
M:F	9 of 9 female
Median age (range)	49 (32–66)
Presentation	
Seroma	5/9 (56%)
Breast swelling/pain	3/9 (33%)
Peri-implant mass	3/9 (33%)
Lymphadenopathy	4/9 (44%)
Confined to seroma	4/9 (44%)
Median time from implantation (years) (range)	10 years (8–29)
Stage IA-IC	4/9 (44%)
T1 N0 M0	1/9 (11%)
T2 N0 M0	3/9 (33%)
T3 N0 M0	0/9 (0%)
Stage IIA (pT4 N0 M0)	1/9 (11%)
Stage IIB (T1-T3 N1 M0)	2/9 (22%)
Stage III (T4 N1-2 M0)	2/9 (22%)
Stage IV (TanyNanyM1)	0/9 (0%)
CD30+	9/9 (100%)
CD3+	1 partial/9 (11%)
CD4+	8/9 (3 partial) (89%)
CD8+	0/9 (0%)
pSTAT3+	1/1 (100%)
CA9+	2/2 (neg in diffuse areas in LN) (100%)
Monoclonal TR	5/5 (100%)
IRF4/DUSP22 rea	0/4 (0%)
P63 rea	0/1 (0%)
Mutations	EPHA3, KMT2A, MALT1, STAT3 (*n* = 2)

*M*, male; *F*, female; *HIV*, human immunodeficiency virus; *LN*, lymph node; *T*, tumor; *N1*, regional lymph nodes; *N2*, non-regional lymph nodes; *M*, metastasis; *rea*, rearrangement; *TR*, T-cell receptor; *VUS*, variant of unknown significance

## Conclusion

The cavity-based lymphoma section of the 2022 EA4HP/SH lymphoma workshop not only confirmed the clinical settings and pathologic characteristics of the established diagnostic entities but also brought about interesting dialog and lively debate of new entities. Within the group of HHV8-positive lymphomas, there are cases with some atypical features that need to be clarified to better define the borders between HHV8+DLBCL and ECPEL and between ECPEL and GLPD. Additionally, consideration of more recent data on the expression of CD138, IgM, and light chains in PEL/ECPEL in upcoming classification schemes will be of interest. HHV8-negative effusion-based lymphomas need further definition and more precise inclusion criteria. Given that the unifying aspect of published studies on HHV8-negative effusion-based lymphoma is the lack of an associated mass (with variable clinical settings and immunophenotype), the existing literature has not been a pure cohort. The effusion-only, HHV8-negative, EBV-positive, and CD20-positive cases that were submitted to the workshop were associated with immunosuppression, indicating that these cases belong either to EBV-positive LPD or EBV-positive DLBCL. Alternatively, the HHV8-negative, EBV-positive, and CD20-negative effusion cases submitted to the workshop had a plasmacytic/plasmablastic phenotype, morphology, and *MYC* rearrangement, indicating that these cases belong to PBL. Following these general criteria, the cases submitted to the workshop could be clearly separated into three groups. The panel recommends to follow these criteria until new studies are available. The submitted FA-DLBCL cases demonstrated there are bonafide EBV-negative cases and cases with plasmablastic morphology and immunophenotype, expanding the definition of the disease. Furthermore, FA-DLBCL can also be present in breast implants, where it should be differentiated from BIA-ALCL.

### Supplementary information

ESM 1 (PDF 276 kb)

## Data Availability

Not applicable.
