# Correction to: Cavity-based lymphomas: challenges and novel concepts. A report of the 2022 EA4HP/SH lymphoma workshop

**DOI:** 10.1007/s00428-023-03664-w

**Published:** 2023-09-25

**Authors:** Arianna Di Napoli, Lori Soma, Leticia Quintanilla‑Martinez, Laurence de Leval, Lorenzo Leoncini, Alberto Zamò, Siok‑Bian Ng, Sarah L. Ondrejka, Fina Climent, Andrew Wotherspoon, Stefan Dirnhofer

**Affiliations:** 1 Department of Clinical and Molecular Medicine, Sant’Andrea University Hospital, Sapienza University of Rome, Rome, Italy; 2 Department of Pathology, City of Hope National Medical Center, Duarte, CA USA; 3 Institute of Pathology and Neuropathology, Eberhard Karls University of Tübingen and Comprehensive Cancer Center, University Hospital Tübingen, Tübingen, Germany; 4 Institute of Pathology, Department of Laboratory Medicine and Pathology, Lausanne University Hospital and Lausanne University, Lausanne, Switzerland; 5 Department of Medical Biotechnology, University of Siena, Siena, Italy; 6 Institute of Pathology, University of Würzburg, Würzburg, Germany; 7 Department of Pathology, Yong Loo Lin School of Medicine, National University of Singapore, Singapore, Singapore; 8 Pathology, and Laboratory Medicine Institute, Cleveland Clinic, Cleveland, OH USA; 9 Pathology Department, Hospital Universitari de Bellvitge, IDIBELL, L’Hospitalet De Llobregat, Barcelona, Spain; 10 Department of Histopathology, The Royal Marsden Hospital, London, UK; 11 Institute of Medical Genetics and Pathology, University Hospital Basel, University of Basel, Basel, Switzerland

**Correction to: Virchows Archiv**

10.1007/s00428-023-03599-2

The article title of the original published version of the above article was incomplete. The correct title is shown as follows:

- **“Cavity‑based lymphomas: challenges and novel concepts. A report of the 2022 EA4HP/SH lymphoma workshop”**

The original article has been corrected.

