# Supplementary material for: Cavity-based lymphomas: challenges and novel concepts. A report of the 2022 EA4HP/SH lymphoma workshop
Source: Virchows Arch. 2023 Aug 9;483(3):299–316. doi: 10.1007/s00428-023-03599-2 (PMC10542738; doi:10.1007/s00428-023-03599-2)
Supplement: Supplementary file 1 — (PDF 276 kb) [file 428_2023_3599_MOESM1_ESM.pdf]

SUPPLEMENTAL TABLES

Supplemental Table 1. EBV positive Primary Effusion Lymphoma (PEL)

Case	Submitter	Panel Diagnosis	Clinical /Pathological/ Molecular Features
1036	L. Chen, Beijing, China	PEL	HIV- male (89 yo), pleural effusion, h/o multicentric Castleman disease (treated with chemotherapy)<br>**Morph**: Immunoblastic<br>**IP:** HHV8+, EBER+, MUM1+, CD20-, CD30+, CD38-, CD138-<br>**FISH:** *BCL2/BCL6/MYC/CCND1* rea-<br>**Mdx:** NGS mutations: *MAML2* p.G838S and *KMT2C* p.G838S. CNV: *CDK4* and *ERBB3* amplification of 4 copies on chromosome 12 with 2.25 Mbs fragment<br>**FU:** Pleural effusion increased, development of pericardial fluid with death in 3 weeks
1050	A. Shestakova, Salt Lake City, UT, USA	PEL	HIV/AIDS+ male (36 yo), pleural effusions, LN with KS<br>**Morph**: plasmablastic/anaplastic<br>**IP:** HHV8+, EBER+, CD3-, CD20 partial+, CD30+, CD138+ (subset), kappa+, lambda-<br>**FU**: 3 cycles of DA-R-EPOCH, intrathecal methotrexate and Brentuximab, Doxorubicin and Paclitaxel for KS. Recurrent KS, hospice and death
1423	O. Padilla, El Paso TX, USA	PEL	HIV/AIDS+ male (33 yo), pleural / pericardial effusions, with KS<br>**Morph**: immunoblastic/plasmablastic<br>**IP:** HHV8+, EBER+, CD3-, CD10-, CD20-, CD30+, CD38+, CD45+, CD56-, lambda+, kappa-<br>**RK**:46-47,X,-Y,+1,+8,add(12)(q24.3),+14,der(1;14)(q12;q22),+mar[cp29]/46,XY[1]

Abbreviations: CNV copy number variation; DA dose adjusted; DHAX dexamethasone, cytarabine, oxaliplatin; EPOCH etoposide, prednisone, vincristine, cyclophosphamide, doxorubicin hydrochloride; FISH fluorescence in situ hybridization; FU follow up; HAART highly active antiretroviral therapy; HIV human immunodeficiency virus; h/o history of; IGH immunoglobulin heavy chain; IP immunophenotype; KS Kaposi Sarcoma; LN lymph node; Mdx molecular analyses; Morph morphology; nml normal; PEL primary effusion lymphoma; R rituximab; RK routine karyotype; rea rearrangement; s/p status post; nml normal; yo year old; yrs years; + positive; - negative.

Supplemental Table 2. Primary Effusion Lymphoma (PEL) with dissemination (2 EBV-positive and 2 EBV-negative)

Case	Submitter	Panel Diagnosis	Clinical /Pathological/ Molecular Features
1038	W. Lin, New York, NY, USA	EBV+ PEL, with dissemination	HIV/AIDS+ (<1 year) male (34 yo), empyema and concurrent tissue / LN involvement<br>**Morph**: immunoblastic/plasmablstic/anaplastic<br>**IP:** HHV8+, EBER+, CD3 dim+, MUM1+, CD10-, CD19-, CD20-, CD30-, CD45-, CD79a-, CD138-, EMA-, IgM-, MYC+/-, light chains-<br>**RK:** complex near triploid<br>**FISH**: *IGH, BCL2, BCL6, MYC* rea-<br>**Mdx:** IGH monoclonal, TRB polyclonal<br>**FU:** HAART and 6 cycles EPOCH + daratumumab with near CR. Retroperitoneal mass 2 months post chemotherapy, started DHAX
1149	T. Bhavsar, Washington, DC, United States	EBV+ PEL with extensive extracavitary dissemination (extracavitary presentation)	HIV+ male (59 yo), h/o syphilis, KS, fever, diffuse LAD, pleural effusion<br>**Morph**: Plasmablastic<br>**IP:** HHV8+, EBER+, CD138 -, CD3-, CD5-, CD10-, CD20-, CD30+ (focal weak/variable), CD45+ (focal weak/variable), CD43+, CD56-, CD117-, BCL6+ (focal weak), IgM+, LC-, PAX5-, MUM1+, MYC+, p53+<br>**Mdx:** IGK, TRG & TRB monoclonal, IGH polyclonal<br>**FISH:** *MYC/BCL2/BCL6* rea- with extra signals
1310	L. Veloza, Lausanne, Switzerland	MPTLD, EBV- PEL, with dissemination	HIV- male (79 yo) s/p renal transplant (12 yrs prior), pleural effusion and colonic involvement.<br>Morph: Plasmablastic<br>**IP:** HHV8+, EBER-, CD3-, CD10-, CD19-, CD20-, CD30+, CD45-/+, CD79a-, CD138-/+ (more expression in colon), EMA+, MUM1+, light chains-, BCL2-, BCL6-, PAX5-<br>**FISH:** *BCL2/BCL6/MYC* rea-<br>**FU**: Daratumumab, developed COVID-19, transferred to palliative care
1216	L. Mescam, Marseille, France	EBV- PEL, with dissemination	HIV- male (66 yo), ascites, concurrent peritoneal involvement and moderately PET avid LN, development of Kaposi while in remission after 1 yr.<br>**Morph**: immunoblastic/plasmablastic<br>**IP:** HHV8+, EBER-, CD2-, CD3+ (variable), CD4+ (variable), CD5-, CD7-, CD8-, CD20-, CD30+, CD45+, CD138+, ALK-, PAX5-, MUM1+, light chains-

			**FISH**: *BCL2/BCL6/MYC* rea-<br>**Mdx:** IGH & TRB monoclonal, TRG polyclonal<br>**FU**: Chemotherapy, complete remission (1 year)

Abbreviations: DHAX dexamethasone, cytarabine, oxaliplatin; EPOCH etoposide, prednisone, vincristine, cyclophosphamide, doxorubicin hydrochloride; FISH fluorescence in situ hybridization; FU follow up; HAART highly active antiretroviral therapy; HIV human immunodeficiency virus; h/o history of; IP immunophenotype; KS Kaposi Sarcoma; LN lymph node; Mdx molecular analyses; Morph morphology; nml normal; PEL primary effusion lymphoma; R rituximab; RK routine karyotype; rea rearrangement; IGH immunoglobulin heavy chain; IGK immunoglobulin kappa light chain; TRG T-cell receptor gamma gene; TRB T-cell receptor beta gene; nml normal; yo year old; + positive; - negative.

Supplemental Table 3. EBV negative Primary Effusion Lymphoma (PEL)

Case	Presenter/Submitter	Panel Diagnosis	Clinical /Pathological/ Molecular Features
1194	P. Barone, New York, NY, USA	PEL	HIV- male (89 yo), pleural effusions<br>**Morph**: immunoblastic/plasmablastic/anaplastic<br>**IP:** HHV8+, EBER-, CD3-, CD10-, CD19-, CD20 dim to -, CD45 variable, CD138+, Blimp1+, BCL6-, PAX5-, IgM-, MUM1+, OCT2-<br>**RK**: Complex<br>**FISH**: *CCND1, BCL2, BCL6* rea-<br>**Mdx**: IGH/IGK monoclonal; NGS *BCL6* mutation, no mutations in *RAS* family or *TP53*; GEP plasmablastic profile<br>**FU**: Chemotherapy, alive after 12 m
1116	S. Sethi, New York, NY, USA	PEL	HIV+ male (54 yo) but stable CD4 count/low viral load, concurrent desmoid tumor, on HAART. IGK MGUS, 9% abnormal PCs in marrow that are CD56+, CD117+, HHV8-.<br>**Morph**: Plasmablastic<br>**IP:** HHV8+, EBER-, CD3-, CD20-, CD30-, CD38+, CD56-, CD117-, CD79a-, CD117-, CD138+, BCMA+, PAX5-, MYC+, light chain negative<br>**FU**: 2 cycles doxorubicin, etoposide, vincristine. Death due to COVID-19.
1120	J. Said, Los Angeles, CA, USA	PEL	HIV/AIDS+ male (33 yo), pleural effusions, h/o Kaposi (skin and LN) and Syphilis.<br>**Morph**: Plasmablastic<br>**IP:** HHV8+, EBER-, CD45+

			**Mdx:** NGS negative (54 gene panel)<br>**FU**: HAART and DA-R-EPOCH
1123	S. Gibson, Scottsdale, AZ, USA	MPTLD, PEL	Male (71 yo), pleural effusion, h/o TPLL s/p HSCT, developed Kaposi's and relapsed TPLL. Ruxolitinib part of immunosuppressive regimen.<br>**Morph**: Plasmablastic<br>**IP:** HHV8+, EBER-, CD2-, CD3-, CD4-, CD5 partial+, CD7 partial+, CD8-, CD20-, CD30+, CD38+, CD45 dim+, CD138+ focal / variable, EMA+ variable<br>**FU**: Alemtuzumab salvage, persistent PEL, hospice, death 4 moths following PEL diagnosis
1201	E. Swenson, Aurora, CA, USA	MPTLD, PEL	Male (42 yo), pericardial effusion, h/o Wegener's granulomatosis, s/p renal transplant (20 yrs prior).<br>**Morph**: Plasmablastic<br>**IP:** HHV8+, EBER-, CD3-, CD20-, CD30+ (strong), CD38+ (bright), CD138+ variable, CD45+, MUM1+, surface light chain-<br>**FU**: Stem cell transplant, multiple infectious complications, hospice and death within 5 months of diagnosis (1 month after transplant)
1108	A. Moreno, Sabadell, Spain	PEL	Male (81 yo), pleural effusion, 2 yrs without therapy before progression.<br>**Morph**: plasmablastic/anaplastic<br>**IP:** HHV8+, EBER-, CD20-, CD30+, CD45+, CD138+, MUM1+<br>**FU**: No chemo, disease stable for 2 yrs, recurrent disease with palliative therapy, died within 3 m

Abbreviations: COVID-19 coronavirus disease of 2019; : DA dose adjusted; EBV Epstein-Barr virus; EPOCH etoposide, prednisone, vincristine, cyclophosphamide, doxorubicin hydrochloride; FISH fluorescence in situ hybridization; FU follow-up; GEP gene expression profile; HAART highly active antiretroviral therapy; HSCT hematopoietic stem cell transplant; h/o history of; IP immunophenotype; LBCL large B cell lymphoma; LC light chain; LN: lymph node; LAD lymphadenopathy; m months; MPTLD monomorphic post-transplant lymphoproliferative disorder; Mdx molecular; MGUS monoclonal gammopathy of uncertain significance; Morph morphology; nml normal; PEL primary effusion lymphoma; R rituximab; rea rearrangement; s/p status post; IGH immunoglobulin heavy chain; IGK immunoglobulin kappa light chain; RK routine karyotype; TPLL T prolymphocytic leukemia; yo year old; yrs years; + positive; - negative.

Supplemental Table 4. Extracavitary Primary Effusion Lymphoma (ECPEL) and associated disorders

Case	Submitter	Panel Diagnosis	Clinical /Pathological/ Molecular Features
1192	A. Dashora, London, United Kingdom	ECPEL	HIV+ male (54 yo) with poor compliance, subcutaneous thigh nodule<br>**Morph** : immunoblastic/plasmablastic<br>**IP:** HHV8+, EBER+, CD45+, CD138+ (heterogeneous), MUM1+, CD3-, CD5-, CD7-, CD10-, CD20-, CD30-, CD45+ (heterogeneous), CD79a, PAX5-, IgM+, kappa+
1196	L. Xerri, Marseille, France	ECPEL	HIV+ (29 yrs) male (47 yo), well controlled, cervical LN<br>**Morph**: Immunoblastic/centroblastic<br>**IP:** HHV8+, EBER+, CD138 -/equivocal, CD2-, CD3-, CD4-, CD5-, CD7-, CD8-, CD20-, MUM1+, CD45 weak+, CD30+, CD56-, CD79a-, IgM-, LC-, PAX5-, MYC+<br>**FISH:** *BCL6* rea+, *BCL2/MYC* rea-<br>**Mdx:** IGK monoclonal, IGH polyclonal, TRB minor clone, TRG polyclonal; Array CGH: dels 1p, 2q, 7p, 14q; gain 15q21-25.
1259	S. Montes-Moreno, Santander, Spain	ECPEL	HIV+ male (62 yo), asymptomatic, cervical LN.<br>**Morph**: Immunoblastic/plasmablastic<br>**IP:** HHV8+, EBER+, CD138+, IgM+, MUM1+, CD3-, CD20-, LC-, CD30-, PAX5-, pSTAT+<br>**FISH:** *MYC* rea -
1297	B. Aqil, Chicago, IL, United States	ECPEL	HIV+ male (50 yo), diffuse LAD, retroperitoneal and perinephric soft tissue masses and nodular thickening of lateral bladder wall (bladder bx)<br>**Morph**: Anaplastic<br>**IP:** HHV8+, EBER+, CD2-, CD5-, CD10-, CD4+, CD30+ (focal), CD79a+ (focal), MUM1+, CD138+, CD20-, focal Kappa, lambda -, ALK-, PAX5-
1063	K. Karube, Nagoya, Japan	HHV8+LBCL vs ECPEL	HIV-, male (81 yo), weight loss, enlarged testicle, kidney mass biopsy<br>**Morph**: Immunoblastic/centroblastic<br>**IP:** HHV8+, EBER-, partial CD3+, CD5-, CD10-, CD30+, CD138+, MUM1+, CD3+ partial, IgM-, IgG-, LC- CD20-, CD79a-, PAX5-, BCL6-, ALK-<br>**FISH:** *MYC* rea-<br>**Mdx:** IGH clonal; TRG polyclonal; *MYD88* wild-type<br>**FU**: refused chemotherapy, moved to palliative care hospice
1143	L. Rimsza, Scottsdale, AZ,	MPTLD, HHV8+LBCL vs	HIV+ male (42 yo) post-transplant with splenomegaly and cytopenias, bone marrow biopsy

	United States	ECPEL	**Morph**: Plasmablastic/anaplastic<br>**IP:** HHV8+, EBER-, CD138+, MUM1+, CD3-, CD5-, CD10-, CD20-, CD30+, CD56-, LC-, BCL1-, BCL6-, BCL2-, IgM-, MYC+<br>**RK:** nml<br>**FU**: Chemotherapy with several complications, death 3 months
1464	A. Serrano, New York, NY, United States	Limited involvement by ECPEL	male (83 yo), h/o urothelial carcinoma, inguinal LN evaluated for possible carcinoma<br>**Morph**: Plasmablastic/anaplastic<br>**IP:** HHV8+, EBER+, CD3+ (weak, subset), CD4 (weak subset), MUM1+, CD38+ (weak), CD43+, CD2-, CD5-, CD7-, CD10-, CD30-, CD45-, CD20-, LC-, PAX5-, CD79a-, CD138-, LMP1-, EBNA2-, PDL1-, OSCAR-<br>**Mdx:** TRG/TRB/IGH/IGK polyclonal<br>**FU**: 3 months (asymptomatic, no PET avid lymph nodes)

Abbreviations: GLPD germinotropic lymphoproliferative disorder; FISH fluorescence in situ hybridization; FU follow-up; h/o history of; IP immunophenotype; ; LAD lymphadenopathy; LBCL large B cell lymphoma; LC light chain; LN lymph node; MPTLD monomorphic post transplant lymphoproliferative disorder; Mdx molecular; Morph morphology; IGH immunoglobulin heavy chain; IGK immunoglobulin kappa light chain; TRG T-cell receptor gamma gene; TRB T-cell receptor beta gene; nml normal; rea rearrangement; RK routine karyotype; yo year old; yrs years; + positive; - negative.

Supplemental Table 5. Reported overall survival in PEL, HHV8 negative EB-LBCL and DLBCL

	**Diagnosis**	**Source/Location**	**Therapy vs None/Drainage alone**	**OS**
WHO-R4	PEL	Not specified	Not specified	Median <6 months
Baidoun et al*	PEL	USA NCDB	Therapy	Median 13 months
Baidoun et al*	PEL	USA NCDB	None/Drainage	Median 3.4 months
Hu et al	PEL/ECPEL	USA based	Both	Median 42.5 months
Alexanian et al	HHV8 neg EBLBCL	Not specified	Both	Median ~10 months
Kaji et al	HHV8 neg EBLBCL	Japanese	Both (87.5% therapy)	84.7% 2-year survival
Gisriel et al	HHV8 neg EBLBCL	Japanese	Both	Median 63.6 months
Gisriel et al	HHV8 neg EBLBCL	Non-Japanese	Both	Median 11 months
Gisriel et al	HHV8 neg	Western	Both	Median 14.9 months

	EBLBCL			
Gisriel et al	HHV8 neg EBLBCL	Western	Chemotherapy	Median 41.2 months; 62% 2-year survival
Gisriel et al	HHV8 neg EBLBCL	Western	None/Drainage	Median 7 months; 33% 2-year survival
WHO-5	DLBCL, NOS	Not specified	Therapy, R-CHOP	~70% 5-year survival
Sehn et al*	DLBCL	Not specified	Therapy, R-CHOP	71-85% 2–3-year survival

Abbreviations: CHOP cyclophosphamide, doxorubicin, vincristine, prednisone; EBLBCL Effusion Based Large B cell lymphoma; HHV8 human herpes virus 8; PEL Primary Effusion Lymphoma; DLBCL diffuse large B cell lymphoma; OS overall survival; NCDB National Cancer Database; USA United States of America

*These articles not cited in the text of the manuscript: Baidoun F, et al. Clinical Characteristics and Survival Outcomes of Primary Effusion Lymphoma: A National Cancer Database Study, *Clin Lymph Myel and Leuk*, 2022;22(7):e485-e494. Sehn LH and Gascoyne RD, Diffuse large B cell lymphoma: optimizing outcome in the context of clinical and biologic heterogeneity, *Blood*, 2015;125(1):22-32.

Supplemental Table 6. Effusion based / fluid overload, HHV8 negative, EBV negative, B cell immunophenotype large B cell lymphoma (EB/FO-LBCL)

Case	Presenter/Submitter	Panel Diagnosis	Clinical /Pathological/ Molecular Features
1065	N. Aggarwal/A.Davis, Pittsburgh, PA, United States	HHV8 negative EB/FO-LBCL	90 yo F, CHF, pericardial and bilateral pleural effusions, mildly prominent mediastinal adenopathy<br>**Morph:** Immunoblastic/plasmablastic/anaplastic<br>**IP:** HHV8-, EBER-, CD19+, CD20+, PAX5+, CD22+, CD79+, CD138+, MUM+, MYC+ (>40%), BCL6+ (weak), lambda+, CD30-, CD3-, CD5-, CD10-, CD38-, CD56-, rare EMA+, BCL1-<br>**Mdx:** >30% VAF: *MYD88, PRDM1, KLHL6, CREBBP*; <10% VAF: *PIM1* (see figure 7 for details)<br>**FISH:** *MYC/BCL2/BCL6* rea-, *IGH::MYC* rea-<br>**FU**: Therapeutic thoracentesis. Palliative care
1193	A. Dashora, London, United Kingdom	HHV8 negative EB/FO-LBCL	74 yo M, Ischemic heart disease and chronic kidney disease, pericardial effusion<br>**Morph:** Anaplastic/immunoblastic<br>**IP:** HHV8-, EBER-, CD20+, MUM1+, CD5-, CD10-, BCL2+, BCL6+, MYC+ (reported double expressor).<br>**FISH:** *MYC* rea-<br>**FU**: R-CHOP, alive 2 yrs after presentation
1198	S. Gisriel, New Haven,	HHV8 negative	80 yo F, HIV-, Heart failure, pleural effusion

	CT, United States	EB/FO-LBCL	**Morph:** Pleomorphic<br>**IP:** HHV8-, EBER-, CD20+, PAX5+, CD45+, lambda+, MUM1+, CD138-, CD3-, CD5-, CD10-, BCL1-, BCL6+, IgM+, MYC+ (40%), BCL2+ (80%), CD38+, CD43-<br>**FISH:** *MYC::BCL2* rea-, *BCL6* rea+<br>**FU**: Supportive care, 2 yr f/u, died with persistent disease
1219	Y. Al-Ghamdi, New York, NY, United States	HHV8 negative EB/FO-LBCL	80 yo M, melanoma, HTN, afib, pleural effusion<br>**Morph:** Immunoblastic<br>**IP:** HHV8-, EBER-, CD20+, PAX5+, BCL6+, MUM1+, CD138-, CD5-, CD10-<br>**FISH:** *BCL6* rea+, *MYC::IGH & IGH::BCL2* rea- (extra copies of *IGH, BCL2, MYC*)<br>**Mdx:** NGS: *MAP2K1* p.Asp67Asn, 17.54%; *CCND3* p.Ile290Arg, 16.97%; *CD58* p.Glu178*, 15.99%; *TNFAIP3* p.Pro457Alafs*16, 10.27%; *NOTCH2* p.Ser2145*, 15.23%; *ZNF292* p.Asp798Asn, 15.7%; *PIK3CA* p.Pro786Ser, 15.15%; *NFKBIA* p.Pro56Ser, 14.92%; *RB1* p.Lys122Glu, 20.5%; *IGLL5* numerous coding and non-coding variants<br>**FU**: Resolved with thoracentesis alone.
1243	A. Volaric, Stanford, CA, United States	HHV8 negative EB/FO-LBCL	82 yo F, CHF, afib, pericardial and pleural effusions<br>**Morph:** Immunoblastic/plasmablastic<br>**IP:** HHV8-, EBER-, CD19+, CD20+, PAX5+, MUM1+, CD138+, BCL6+, BCL2+, MYC+ (>40%) CD3-, CD5-, CD10-, CD30-, CD38-, CD56-, CD117-, BCL1-, SOX11-<br>**FISH:** *BCL6* rea+, *MYC::BCL2* rea-<br>**FU**: One cycle mini R-CHOP.
1412	S. Kohla, Doha, Qatar	HHV8 negative EB/FO-LBCL	91 yo M, HIV-, HTN, cardiomyopathy, kidney failure, pleural and pericardial effusion<br>**Morph:** Immunoblastic/plasmablastic/anaplastic<br>**IP:** HHV8-, EBER-, CD19+, CD20+, CD45+, IgM+, dim lambda+, partial CD38+, CD5-, CD10-, CD43-<br>**FU**: Palliative therapy
1419	A. Nicolae, Strasbourg, France	HHV8 negative EB/FO-LBCL	77 yo F, HIV-, Hypoalbuminemia, pericardial and pleural effusions<br>**Morph:** Immunoblastic/plasmablastic/anaplastic<br>**IP:** HHV8-, EBER-, CD10+, CD79a+, CD20+, PAX5+,

			MUM1+, CD10+, BCL6+, BCL6+, CD45+, CD138-, CD5-, Myc-, PDL1-, CD3-, CD30-<br>**Mdx**: IGH monoclonal<br>**FU**: R-mini-CHOP, CR 6 months f/u.

Abbreviations: afib atrial fibrillation; CHF congestive heart failure; CHOP cyclophosphamide, doxorubicin, vincristine, prednisone; CR complete remission; EB-LBCL effusion based large B cell lymphoma; EBV Epstein-Barr virus; FU follow up; FISH fluorescence in situ hybridization; HHV8 human herpes virus 8; HTN hypertension; IP immunophenotype; IGH immunoglobulin heavy chain; LC light chain; M male; Mdx molecular; nml normal; rea rearrangement; R rituximab; RK routine karyotype; VAF variant allele frequency; yr year old; yr year; + positive; - negative.

Supplemental Table 7. HHV8 negative , EBV positive, large B cell lymphoma (LBCL) effusion based / presenting as an effusion

Case	Submitter	Panel Diagnosis	Clinical /Pathological/ Molecular Features
1161	L. Veloza, Lausanne, Switzerland	EBV+ LBCL, presenting as effusion, effusion only	40 yo M with CML, treated with Dasatinib for ~2 yrs, developed pericardial effusion without mass<br>**Morph:** Immunoblastic/plasmablastic<br>**IP:** HHV8-, EBER+, CD20+, MUM-/+, CD10-, LMP1+<br>**FISH:** *MYC/BCL2/BCL6* rea-<br>**Mdx***:* IGH monoclonal; NGS: variants of uncertain significance, *BRAF* exon 17: c.2127+7A>G (splicing site) (VAF 46%) and *NOTCH2* exon 26: c.4537T>C (p.Phe1513Leu) (VAF 30%); RT-MLPA unclassified molecular cell of origin subtype<br>**FU**: Changed to Nilotinib and 4 cycles R-CHOP with CR (3 months f/u).
1088	M. Chiselite, Grand Rapids, MI, United states	EBV+ LBCL, presenting as effusion, effusion only	46 yo M with prior pneumonectomy 14 yrs for carcinoid tumor, ulcerative colitis treated with prednisone, and reported history of immunotherapy for chronic rhinitis; pleural effusion without reported mass<br>**Morph:** Immunoblastic/plasmablastic/anaplastic<br>**IP:** HHV8-, EBER+, CD20+, CD30+, PAX5+, CD43+, CD2-, CD3-, CD4-, CD8-, EMA-, ALK-
1075	K. Rech, Rochester, MN, United States	EBV+ LBCL presenting as an effusion	64-yo M; history of emphysema, diastolic heart failure and diabetes, bilateral pleural effusions, PET scan with mild uptake (SUV 3.4-4.5) in left lateral lower lobe pleura, left intercostal space, left subcutaneous region, right lateral lower lobe pleura and right supraclavicular lymph nodes

			**Morph**: Non-invasive large atypical lymphoid cells within fibrin on the inner surface of fibrotic pleural biopsy.<br>**IP:** CD20+, PAX5+ (weak), CD45+, non-GCB (CD10-, BCL6-, MUM1+), CD138+ (weak), CD30+(weak), BCL2+, MYC+, LMP1+, EBER+, CD3-, HHV8-<br>**Mdx**: EBV PCR+ in PB

Abbreviations: CHOP cyclophosphamide, doxorubicin, vincristine, prednisone; CR complete response; FU follow up; FISH fluorescence in situ hybridization; HHV8 human herpes virus 8; IP immunophenotype; LBCL large B cell lymphoma; R rituximab; M male; Mdx molecular; nml normal; rea rearrangement; IGH immunoglobulin heavy chain; PB peripheral blood; PCR polymerase chain reaction; RK routine karyotype; RT-MLPA reverse transcriptase multiplex ligation-dependent probe amplification; VAF variant allele frequency; yr year old; yrs years; + positive; - negative

Supplemental Table 8. Plasmablastic lymphoma (PBL) presenting as an effusion

Case	Presenter / Submitter	Panel Diagnosis	Clinical /Pathological/ Molecular Features
1382	B. Mai/W. Wang, Houston, TX, USA	MPTLD, PBL presenting as an effusion	63 yo M, cirrhosis, HCV+, s/p liver transplant 11 yrs prior. Ascites<br>**Morph**: Plasmablastic/pleomorphic morphology<br>**IP:** HHV8-, EBER+, MUM1+, EMA+, weak CD45+, CD20-, CD138+flow/-IHC, CD38+, CD2-, CD3-, CD5-, CD7-, PAX5-,<br>**RK:** 83-89<4n>,XXY, +X,der(X)t(X;1)(q22;p22)x2,+1,der(1;9)(q10;q10),dic(1;22)(p12;q13), add(3)(p12),-4,add(4)(q31),t(8;14)(q24;q32)x2,-10,-13,del(13)(q12;q22),-14,der(14)t(8;14), der(16), t(1;16)(q21;q12)-18,+1-3mar[cp8]/46,XY[12].<br>**FISH**: *MYC::IGH* rea+<br>**FU**: 3 cycles DA-CHOP, death within 3 months
1055	D. Jevremovic , Rochester, MN, USA	PBL presenting as an effusion	77 yo M, ETOH cirrhosis with long-standing ascites and increased need for therapeutic thoracentesis; BM- for plasma cell neoplasm, SPEP/IFIX-<br>**Morph**: Plasmablastic/plasmacytoid and mature plasma cells<br>**IP:** HHV8-, EBER+, CD138+, MUM1+, lambda+, CD20-, LMP1-, CD3-, CD30-, kappa-<br>**FISH:** *MYC* rea+, *BCL2/BCL6* rea-, 3-5 copies of *BCL2* and *BCL6*<br>**FU**: Patient did well (3 yr f/u with apparent response to 1 course Val/Dex).
1426	K. Miller,	PBL presenting	50 yo M, HTN, DVT and PE, pericardial effusion

	Baltimore, MD, USA	as an effusion	**Morph**: Immunoblastic/Plasmablastic/Pleomorphic morphology<br>**IP:** HHV8-, EBER+, CD30+, CD38+, MUM1+; rare-partial expression of CD3, CD15, CD138, CD4 and CD56 (dim); CD20-, CD2-, CD5-, PAX5-, CD79a-, lambda-, kappa-, OCT2-, ALK1-<br>**FISH***: MYC* rea+, *BCL2/BCL6* rea-<br>**Mdx:** IGH and TRG monoclonal
1424	O. Padilla, El Paso, TX, USA	PBL presenting as effusion and retroperitoneal mass	59 yo M, HIV+, chronic renal failure, diabetes, presenting as bilateral pleural effusions, and retroperitoneal mass/adenopathy.<br>**Morph**: Plasmacytoid/Plasmablastic morphology<br>**IP:** HHV8-, EBER+, CD138+, CD56+, CD3-, CD20-, MUM1-, lambda+, kappa-, CD5-, CD10-, CD79a-
1357	E. Quiros, Barcelona, Spain	MPTLD, PBL presenting as an effusion	59 yo F, 9 yrs post renal transplant, presenting with real dysfunction and ascites (with omental/tissue involvement)<br>**Morph**: Plasmacytoid/Plasmablastic morphology<br>**IP:** HHV8-, EBER-, CD79a+, OCT2+, MUM1+, EMA+, CD4+, CD20-, CD138-, PAX5-, CD56-, CD2-, CD3-, ALK1-, BCL1-<br>**FISH***: MYC* rea+<br>**Mdx:** *POT1, TP53, XPO1*<br>**FU:** Rapid deterioration with death in a few days

Abbreviations: HTN hypertension, DVT deep venous thrombosis, PE pulmonary embolism, CHOP cyclophosphamide, doxorubicin, vincristine, prednisone; DA dose adjusted; EBV Epstein-Barr virus; FU follow up; FISH fluorescence in situ hybridization; HHV8 human herpes virus 8; IFIX immunofixation; Morph morphology; IP immunophenotype; PBL plasmablastic lymphoma; M male; Mdx molecular; Morph morphology; SPEP serum protein electrophoresis; IGH immunoglobulin heavy chain; TRG T-cell receptor gamma gene; MPTLD monomorphic post-transplant lymphoproliferative disorder; rea rearrangement; RK routine karyotype; VAF variant allele frequency; yr year old; yrs years; + positive; - negative

Supplemental Table 9. Other lymphomas presenting as an effusion

Case	Submitter	Panel Diagnosis	Clinical /Pathological/ Molecular Features
1053	P. Bulterys, Stanford, CA, USA	ALCL, ALK+	Pediatric patient, F, presenting as pleural effusions with lytic lesion<br>**Morph:** Anaplastic<br>**IP:** CD4+, CD43+, CD30+, CD33+, CD2+ (minor subset), CD7+; CD3-<br>**RK:** t(2;5), del(5q), add(4q)
1208	S. Yang, New York, NY, USA	ALCL, ALK-	45 yo M, presenting with pleural effusions, lung nodules, adenopathy<br>**Morph:** Anaplastic<br>**IP:** CD2+, CD7+, cCD3+, CD30; sCD3-, CD4-, CD8-

1379	E. Mason, Nashville, TN, USA	ALCL, ALK-	65 yo M presented with ascites and multiple masses/adenopathy (presumed to be NSCCA), s/p post checkpoint Inhibitor therapy for NSCCA<br>**Morph:** Anaplastic<br>**IP:** CD3+ (dim, subset), CD4+ (dim), CD43+, CD30+, CD45+ (dim), granzyme B+; CD5-, CD7-, CD8-
1186	T. Shet, Mumbai, India	DLBCL, NOS	56 yo M, h/o CD20- B cell NHL, presenting with effusions and masses/adenopathy<br>**Morph:** Immunoblastic/anaplastic<br>**IP:** CD10+. CD19+, CD30+, CD38+, CD45+, MUM+, OCT+, PAX5+, CD38+, BCL2+, CD319+, CD28+, cytoplasmic Kappa+; CD20-, CD138-, EBER-, HHV8-, HLA-DR-<br>**RK**: 46~48,XY,del(1)(q25),+der(1)del(1)(p32)dup(q32q44),del(1)(p10),i(1)(q10),add(3)(p26),dup(4)(q12q21),+7,add(7)(p22),t(8;22)(q24;q11),del(10)(q22),der(15)t(5;15)(q13;q26.3),add(18)(q23)cp[20] showing t(8;22)(q24;q11) (IGL::MYC), structural abnormalities of chromosomes 1, 4, 15 and trisomy 7

Abbreviations: ALCL anaplastic large cell lymphoma; BV brentuximab vedotin; DLBCL diffuse large B cell lymphoma not otherwise specified; f female; FISH fluorescence in situ hybridization; h/o history of; IP immunophenotype; M male; NHL non- Hodgkin lymphoma; NSCCA non-small cell carcinoma; RK routine karyotype; yo year old; + positive; - negative

Supplemental Table 10. Fibrin associated diffuse large B cell lymphoma (FA-DLBCL) submitted cases

Case ID	Presenter /Submitter	Panel Diagnosis	Site	Clinical /Pathological/ Molecular Features
1032	A. Perry, Ann Arbor, MI, United States	FA-DLBCL	Left atrial myxoma	60-yo M; incidental large (5 cm) left atrial mass on echocardiogram<br>**Morph**: Fibrin clusters and sheets of medium-to-large atypical lymphoid cells<br>**IP:** CD20+, PAX5+, CD45+, GCB (CD10+, BCL6+, MUM1+), BCL2+/-(weak), MYC- (10%), CD3-, CD5-, cyclin D1-, CD30-, TdT-, HHV8-, LMP1-, EBNA2-, EBER-, Ki67= 80-90%<br>**FISH:** *MYC/BCL2/BCL6 rea-*<br>**Mdx:** NGS: mutations in *CD58, HLA-B, CREBBP, HIST1H1E, HIST1H2BD, HIST1H1D; PAX5, CARD11,*

				IKZF3; CD79B, BCL11A, NOTCH1 and *PIM1*<br>**FU:** PET negative, no adjuvant chemotherapy
1384	C. Padrão, Amadora, Portugal	FA-DLBCL	Left atrial myxoma	76-yo F, dizziness and exertional dyspnea, diabetes mellitus type 2, hypercholesterolemia and hypertension, 28x10 mm left atrial hyperechogenic mass<br>**Morph**: Sheet of medium-to-large lymphoid cells with evident nucleoli at myxoma periphery<br>**IP:** CD20+, GCB (CD10+, BCL6+, MUM-1+), BCL2+, MYC+, CD3-, CD43-, cyclin-D1-, EBER-, Ki67=100%<br>**FISH**: *MYC/BCL2/BCL6* rea-<br>**FU:** No other therapies after surgical excision. NED after 11 months
1107	H. Sidhu, Johnson City, NY, U.S.	FA-DLBCL	Left atrial myxoma	50-yo F, fatigue, dyspnea and palpitations, atrial fibrillation, 6 cm left atrial mass<br>**Morph**: Large atypical lymphoid cells with prominent nucleoli on the myxoma surface.<br>**IP:** CD20+, CD79a+, non-GCB (CD10-, BCL6+, MUM1+), BCL2+, MYC+, CD3-, CD5-, CD30-, BCL1-, EMA-, LMP1-, EBER-, PD-L1-, p53+, Ki67 =99%<br>**FISH:** BCL6 rea+ and/or partial deletion; extra copies of *MYC*; gain of all or part of chr 8 and of chr 14 (probable *IGH::*non-*MYC* rea)<br>**Mdx:** IGH mono*clonal;* NGS (128 -genes): *PIM1* c.607+1G>T (VAF 38%), *ETV6* c.367 C>T (VAF 43%); VUS in *BCOR, ETV6, FAT1, HIST1H1E, PIM1*, and *TBL1XR1*; MS PCR: *MGMT* gene promoter methylated at 75.9%<br>**FU:** No other therapies after surgical excision. NED after 24 yrs

1162	M. Barouqa, Rochester, MN, United States	FA-LBCL	Trombus in abdominal aortic aneurysm	74-yo M; uncontrolled hypertension and hyperlipidemia, vague abdominal and flank pain for six months for a rapidly expanding abdominal aortic aneurysm and a densely adherent, inflamed phlegmon along the posterior aspect of the perivisceral aorta<br>**Morph**: Fibrinoid material with atypical lymphoid cells<br>**IP:** CD20+, PAX5+, CD45+, non-GCB (CD10-, BCL6+/-, MUM-/+), MYC+ (80%), BCL2+/-, CD30+/-(partial), CD138-, EBER+, Ki67=90%<br>**FISH:** *MYC/BCL2* rea-<br>**FU:** BM biopsy negative; CR after six cycles of R-CHOP; LBCL GCB-type EBV- neg 5yrs later
1166	R. Morse, Seattle, WA, United States	FA-LBCL	Trombus in aortic graft and left femoral artery	72-yo M; abdominal aortic aneurysm repair graft 6 months before; history of peripheral vascular and coronary artery disease requiring multiple thrombectomies, methotrexate treatment for psoriasis, and diabetes<br>**Morph**: Fibrin and large atypical lymphoid cells in femoral and aortic thrombus<br>**IP:** CD20+, CD45+, non-GCB (CD10-, BCL6+, MUM1+), BCL2+ (50%), MYC- (10%), CD3-, CD34-, TdT-, EBER+, Ki-67=70-90%<br>**FISH:** *MYC/BCL2/BCL6* rea- with extra copies<br>**FU**: BM biopsy negative; Progressive neurological symptoms with multifocal FDG uptake in the CNS and within the abdominal aorta 6 mo later. Treated with R-CHOP

1205	J. Cannatella, Omaha, NE, United States	FA-LBCL	Thrombus in aortic Endurant endograft (composed of a nickel titanium alloy stent with multifilament polyester graft fabric)	64-yo M. Ischemic cardiomyopathy and endovascular abdominal aortic aneurysm repair with Endurant endograft 6yrs before<br>**Morph**: coagulative necrosis and fibrin clot with foci of large atypical lymphoid cells.<br>**IP:** CD20+, CD19+, PAX5+/-(weak), CD79a+(weak), non-GCB (CD10-, BCL6-, MUM1+), GCET1-, FOXP1+(weak), BCL2+, MYC+ (50%), CD3-, CD5-, CD30-, ALK-, EBER+(weak), HHV8-, Ki67=75%<br>**FU:** PET positive one month post diagnosis, BM negative, received R-CHOP with resolution on PET 3 months later (presumed CR)
1229	T. Lee, Los Angeles, CA, United States	FA-LBCL	Multiple thrombus involving bilateral pulmonary artery and graft	30-yo F; congenital heart disease (pulmonary atresia/ventricular septal defect) surgically managed with VSD patch closure and placement of a RVPA homograft connected to a Gore-Tex conduit graft at age 7<br>**Morph**: Fibrin and pleomorphic large atypical lymphoid cells in the graft material and pulmonary thrombus<br>**IP:** CD20+/-(subset), CD30+/-(variable), CD79a+, PAX5+, non-GCB (CD10-, BCL6-, MUM1+), BCL2+ (weak), MYC+(20-30%), CD3-, CD5-, CD31-, CD34-, Cyclin D1-, p53-(1-10%), PD-L1+, EBER+, Ki67 >90%<br>**FU:** R-CVP treatment; left lingular mass after 2mo (presumed embolic involvement); the patient passed away 3mo later (uncertain cause)

1365	C. Williams, Los Angeles, CA, United States	FA-LBCL	Infrarenal abdominal aortic dissecting aneurysm	76-yo M; previous coronary artery bypass graft for coronary artery disease, hypertension, chronic obstructive pulmonary disease, infrarenal abdominal aortic aneurysm for 4 years, presenting with flank pain for aneurysmal dissection of the abdominal aorta<br>**Morph**: Vessel wall and periaortic fibromuscular soft tissue with foci of fibrin and pleomorphic atypical lymphoid cells<br>**IP:** CD20+, CD79a+/-(partial), PAX5+, CD45+(variable), non-GCB (CD10-, BCL6+, MUM1+), CD3+, CD30+/-(variable), CD2-, CD4-, CD5-, CD7-, CD8-, CD15-, CD56-, ALK-, EMA, EBER+<br>**FU:** Patient died shortly after surgery due to complications (multiorgan failure, septic shock, and coagulopathy)
1288	M. Vasef, Albuquerque, NM, United States	FA-LBCL	Cardiac thrombus associated with Melody valve	23-yo M; history of Tetralogy of Fallot (transannular patch, multiple right ventricle to pulmonary artery conduits, Melody valve placement, biventricular pacemaker)<br>**Morph**: Fibrin and pleomorphic large atypical lymphoid cells in the thrombus<br>**IP:** CD20+, CD45+, non-GCB (CD10-, BCL6-, MUM1+), BCL2+, MYC+(50%), CD3-, CD5-, TdT-, CD34-, CD30-, CD138-, ALK-, EBER+, HHV8-, KI67=90%<br>**FISH:** *MYC/BCL2/BCL6* rea- with extra copies<br>**FU:** Patient died for additional thrombotic events and refractory sepsis

1342	H. Pan, Los Angeles, CA, United States	FA-LBCL	Mitral valve with vegetations and multiple cerebral emboli	67-yo M; mitral valve replacement 8yrs before; multiple rim-enhancing lesions involving basal ganglia, temporal lobes, and left posterior frontal lobe at MRI, and. vegetations on the mitral valve leaflet at transthoracic echocardiogram<br>**Morph**: Mitral valve and right temporal lobe brain biopsy tissue with fibrin and large pleomorphic atypical lymphoid cells<br>**IP:** CD20-, PAX5+, CD45+, CD10-, BCL6-(<30%), MUM1+, OCT2+, MYC+(60%), CD3-, CD30+, ALK-, EBER+, HHV8-, Ki67>90%<br>**FU:** Patient's died two weeks after the brain biopsy for brain lesions worsening and severe electrolyte disturbances
1277	S. Ondrejka, Cleveland, OH, U.S.	FA-DLBCL	Left adrenal gland cystic mass (max diam. 15 cm)	70-yo M; left-sided total nephrectomy for renal cell carcinoma 4yrs before<br>**Morph**: Hemorrhage, fibrin and atypical intermediate-sized lymphoid cells.<br>**IP:** CD20+, non-GCB (CD10-, BCL6-, MUM1+), BCL2+, MYC+(40%), cyclin D1-, CD30-, TdT-, EBER+, PD-L1+, IDO1+, CD200+/-(subset), VISTA-, TIM-3-, and LAG-3-, Ki-67= 90%<br>**FU:** Surgical excision only. NED after 6 yrs
1391	H. Hov, Trondheim, Norway	FA-DLBCL	Right adrenal gland cyst	67-yo F, 7 cm right adrenal gland cyst grown in 12yrs<br>**Morph**: Cyst filled with fibrinoid material and medium sized cells with single eosinophilic nucleolus<br>**IP:** CD20+/-(weak and variable), PAX5+, CD79a+, CD45-/+(weak), non-GCB (CD10-, BCL6-, MUM1+), CD138+/-(variable), Kappa+, Lambda-, BCL2+, CD30+, CD23+, EBER+, CD3-, CD5-, CD56-, CD68-, CD34-, cyclinD1-, ALK-, TdT-, MPO-, HHV8-, Ki67=90%

1122	F. Fend, Tübingen, Germany	FA-DLBCL	Cystic spaces within PDGFRA ^{D842V}-mutated gastrointestinal stromal tumor (GIST)	51-yo M; neo-adjuvant treatment with avapritinib for GIST mass<br>**Morph**: Cystic spaces containing fibrinous exudate and medium-to-large atypical cells with prominent nucleoli and basophilic cytoplasm<br>**IP:** CD45-, CD19-, CD20-, CD79a-, PAX5-, CD138+, MUM1+, lambda+, IgA-, MYC+(80%), CD3-, ALK-, TdT-, HHV8-, EBER-, EMA-, CD56-(rare +), CD30-(rare +), Ki-67=90%<br>**FISH:** *MYC* rea+<br>**Mdx:** NGS (78 genes): mutations in *KMT2D, MEF2B, BTG1 and CXCR4*<br>**FU:** LN and BM negative for lymphoma. CR after surgery (1 year follow up)
1363	G. Ippoliti, Barcelona, Spain	FA-DLBCL	Right lobe hepatic cyst	61-yo F, gradual onset of fever, abdominal pain and hepatomegaly, multiple hepatic cysts in right lobe, largest cyst (15.7 x 13.7 cm) removed<br>**Morph**: Fibrinous deposition on inner wall containing large lymphoid cells with conspicuous nucleoli<br>**IP:** CD20+, CD79a+, PAX5+, non-GCB (CD10-, BCL6-, MUM1+), BCL2+/-(focally), MYC-, CD3-, CD5-, CD30+/-(focally), EBER+, HHV8-, Cyclin D1-, Ki67 >90%<br>**Mdx:** IGH monoclonal<br>**FISH:** *MYC/BCL2/BCL6* rea-

1307	J. Goodlad / G. Horne, Glasgow, United Kingdom	FA-DLBCL	Cardiac pacemaker pocket	72-yo M; hemoserous fluid leak following a cardiac pacemaker revision that occurred 4-weeks previously; rheumatic polymyalgia treated with corticosteroids over prolonged and intermittent periods<br>**Morph**: pacemaker pseudocapsule lined by fibrin and full thickness infiltrated by pleomorphic lymphoid cells with plasmablastic morphology.<br>**IP:** CD20-, CD79a-, PAX5-, CD138+, MUM-1+, Lambda+, Kappa-, EBER+, CD10-, BCL6-, BCL2-, CD3-, CD5-, CD30-, CD56-, Cyclin D1-, ALK1-, HHV8-<br>**FU:** CR since 6 yrs after 3 cycles of R-CHOP and local radiotherapy
1257	R. Leguit, Utrecht, Netherlands	FA-DLBCL	Breast Implant	63-yo F; breast implant removal and capsulectomy after 10yrs for capsular fibrosis and calcification<br>**Morph**: Superficial fibrinous exudate with foci of large pleomorphic cells.<br>**IP:** CD20+, CD79a+, PAX5+, CD19+, CD45+, CD10 n/a, BCL6-, MUM1+, BCL2+, MYC+, kappa-, lambda-, p53+, CD30+, EBV LMP1+, EBV EBNA+, EBER+, CD2-, CD3-, CD4-, CD5-, CD7-, CD8-, ALK1-, CD43+, CD56-, HHV8-, granzyme B-, Ki67>80%<br>**FISH:** *MYC/BCL2/BCL6* rea- with *MYC* extra copies<br>**Mdx:** NGS (64 genes): No mutations<br>**FU:** no additional treatment after surgery, NED after 9 months
1290	S. Morgan, Sheffield, United Kingdom	FA-DLBCL	Breast Implant	Persistent seroma at right breast 18 months after mastectomy and axillary clearance for breast cancer<br>**Morph**: Fibrin and large cells with prominent nucleoli infiltrating the inner fibrous capsule<br>**IP:** CD20+, CD79a+, PAX5+/-(patchy), CD45+, OCT2+/-(patchy), CD10 n/a, BCL6-, MUM1+, CD138+, CD23+, Cyclin D1-, CD3-, CD5-, CD30+, EBER+, HHV8-, ALK1-, Ki67=70-80%

1338	S. Rodríguez Pinilla, Madrid, Spain	FA-DLBCL	Breast Implant	55 yo F; pain in the external side of her right breast; cosmetic breast augmentation 15yrs before<br>**Morph**: Atypical large cells immersed within fibrin layer on the inner part of the capsule.<br>**IP:** CD20+, PAX5+, CD30+, LMP1+, EBNA-2+, EBER+, CD3-, HHV8-
1378	E. Poullot, Créteil, France	FA-DLBCL	Breast Implant	56 yo F; bilateral cosmetic macrotextured breast prothesis 26yrs before; axillary lymph nodes excision and bilateral replacement of breast implants (microtextured) with left capsulectomy for HER2-positive left breast cancer treated by neoadjuvant chemotherapy and Trastuzumab; history of melanoma on left shoulder<br>**Morph**: Left side capsule with fibrinoid material, necrosis and large pleomorphic cells<br>**IP:** CD20+, CD79a+, PAX5+, CD19+, non-GCB (CD10-, MUM1+), BCL2+, MYC-, kappa-, lambda-, CD2-, CD3-, CD5-, CD7-, CD4-, CD8-, CD30+, LMP1+, EBNA2+, EBER+, HHV8-, pSTAT3-, ALK1-. Ki67>70%)<br>**Mdx:** IGH monoclonal, TRG polyclonal; NGS (36 genes): no mutations
1097	T. Tousseyn, Leuven, Belgium	FA-DLBCL	Breast Implant	45 yo, HIV+ (under control with therapy); fluctuating left breast swelling for 2 years and night sweats; moderately hypermetabolic lymphadenopathy left axilla (SUV max 4.1); cosmetic bilateral breast implants 9yrs before<br>**Morph:** large pleomorphic cells within fibrinous exudate in the seroma and on the inner part of the capsule without infiltration<br>**IP:** CD20+, CD19+, CD79A+, PAX5+/-(weak/partial), BOB1+, OCT2+, CD23+, MYC+(>30%), CD2-, CD3-, CD5-, CD4-, CD8-, perforin-, TIA1-, granzymeB-, CD30+, CD15-, EBER+, LMP1+, EBNA2+, HHV8-, PD-L1+, Ki67>90%

Abbreviations: yo year old; yrs years; mo months; LN: lymph node; Morph morphology; IP immunophenotype; + positive; - negative; FISH fluorescence in situ hybridization; Mdx molecular analyses; IGH immunoglobulin heavy chain; TRG T-cell receptor gamma gene; NGS next generation sequencing; VAF variant allele frequency; VUS variant of unknown

significance; FU: follow up; NED no evidence of disease; CR complete remission; R-CHOP rituximab cyclophosphamide doxorubicin hydrochloride vincristine sulfate prednisone; CNS central nervous system; FDG 18F-fluorodeoxyglucose.

Table 11. Breast implant associated anaplastic large cell lymphoma (BIA-ALCL) submitted cases

Case ID	Submitter	Panel Diagnosis	Clinical /Pathological/ Molecular Features
1098	Y. Bühler, Zürich, Switzerland	BIA-ALCL	32yo F; seroma, peri-implant nodule, enlarged left axillary, supraclavicular and cervical LN; textured breast implants 8yrs before, with suspected implant rupture for 3 years<br>**Morph**: Large, pleomorphic cells in LN, seroma and mass (pT4, N2)<br>**IP**: CD30+, IRF4+, CD4+, Granzyme B+, Perforin+, TIA1+, PD-L1+, CD25+, CD43+, TCRbeta+, TCRdelta-, Pax5-, CD45-, CD20-, CD79a-, OCT2-, BOB1-, CD2-, CD3-, CD5-, CD7-, CD8-, CD15-/+(partial, faint), ALK-, GATA3-, PD1-, EBER-<br>**Mdx:** prominent peak on polyclonal background of TRG*;* NGS (59 genes): *NOTCH2* VUS p.A3F (VAF 22.7%)
1117	A. Mozos, Barcelona, Spain	BIA-ALCL	43yo F; left breast swelling and homolateral axillary and retro pectoral LN; bilateral breast implants for cosmetic augmentation 10yrs before.<br>**Morph**: Large, pleomorphic cells in axillary LN, seroma and capsule (pT2, N2)<br>**IP:** CD30+, TIA1+, granzyme B+, CD4-/+(weak), ALK-, CD2-, CD3-, CD5-, CD7-, CD8-, CD20-, CD79a- and PAX5-, Ki67>80%<br>**Mdx:** TRB monoclonal<br>**FU:** In CR after 6 cycles of CHOP therapy
1454	P. Dartigues, Villejuif, France	BIA-ALCL	56yo F; seroma, mass and homolateral enlarged axillary LN; breast cancer in Cowden's syndrome with bilateral textured prosthetic reconstruction 10yrs before<br>**Morph**: Large pleomorphic cells in the mass biopsy (pT4, N1)<br>**IP:** CD45+, CD30+, ALK1-, CD15-, MUM1+, EMA-/+(rare), pSTAT3+, CD43+, CD3-, CD5+, CD2-, CD7-, CD4+, CD8-, CD20-, PAX5-, EBER-, CK-, Ki67= 90%<br>**FISH:** *IRF4/DUSP22* rea-

1361	M. Takeda, Los Angeles, CA, United States	BIA-ALCL	51yo F; unilateral seroma; silicone prosthesis 10yrs before<br>**Morph**: Large, pleomorphic cells infiltrating the capsule (pT2)<br>**IP:** CD30+, CD45+, granzyme B+, perforin-/+(rare), TIA1-, MUM1+, ALK1-, CD3-, CD2-, CD4-, CD8-, CD43-, OCT2-, CD138-, CD56-, CD20-, PAX5-. Ki-67 =60-70%
1459	R. Rolim, Lisboa, Portugal	BIA-ALCL	44 yo F; seroma, thoracic mass, intrathoracic lymph nodes; breast cancer with prosthetic reconstruction 20yrs before<br>**Morph**: large pleomorphic cells in the mass and lining but not infiltrating the fibrous capsule (pT4). Foreign body reaction (CD30-) in the LN (pT4 N0)<br>**IP:** CD30+, CD4+, TIA+, granzymeB+/-(focal), ALK-, p63-, CD3-, CD2-, CD5-, CD7-, CD8-, EMA-, CD20-, EBER-<br>**Mdx:** TRG monoclonal<br>**FISH:** *IRF4/DUSP22* rea-<br>**FU:** Salvage therapy after CHOP treatment due to disease progression (de novo intrathoracic adenopathy and adrenal gland involvement by PET scan)
1124	A. Feldman, Rochester, MN, United States	BIA-ALCL	57-yo F; 8-month history of right axillary LN (largest, 5.4 cm); bilateral textured breast implants 29yrs before<br>**Morph**: Large pleomorphic cells in the LN with minimal capsular infiltration (pT2, N1)<br>**IP:** CD45+, CD30+, CD4+/-, granzyme B+/-, CA9+/-(strong in capsule and focal tumor islands in LN and negative in the diffuse tumor cell LN infiltrate), CD2-, CD3-, CD5-, CD7-, CD8-, CD15-, CD20-, CD43-, CD56-, PAX5-, p63-, TIA-1-, ALK-, HHV8-, EBER-, Ki67=90%<br>**FISH:** *DUSP22* rea- with extra copies
1333	A. Domingo, Barcelona, Spain	BIA-ALCL	47-yo F; right-sided recurrent breast swelling; bilateral cosmetic silicone breast implants 9yrs before<br>**Morph**: Large pleomorphic cells in the seroma and lining but not infiltrating the fibrous capsule (pT1)<br>**IP:** CD30+, CD4+, TIA-1+, CD3+/-(focal), CD2+/-(focal), CD7+/-(focal), CD5-, CD8-, ALK-, EMA+/-(focal); CA9+, CD20-, CD79a-, EBER-<br>**Mdx:** TRG monoclonal<br>**FISH:** *DUSP22* and *TP63* rea-

1244	J. Zhou, Indianapolis, IN, United States	BIA-ALCL	66-yo F; left breast pain, warmth, redness, and asymmetry; bilateral breast implants 26yrs before<br>**Morph**: Large pleomorphic cells infiltrating the left capsule (pT2)<br>**IP:** CD30+, CD15+/-(partial), CD5+, CD4+/-(weak), granzyme B+, perforin+, CD43+; ALK-, CD2-, CD3-, CD7-, CD8-, TIA1-, CD20-, PAX5-, CD163-, CD45RB-, cytokeratin AE1/AE3-, EBER-. Ki-67= 80%<br>**Mdx**: TRG monoclonal
1428	R. Auclair, New York, NY, United States	BIA-ALCL	49-yo F, seroma and intermittent red rash on left breast with underlying soreness and fever; sarcoidosis and right breast cancer with bilateral textured prosthetic reconstruction 15yrs before (right) and 10yrs before (left)<br>**Morph**: Large pleomorphic cells in effusion and infiltrating the capsule (pT2)<br>**IP:** CD30+, CD4+, CD45+, CD3-, CD2-, CD5-, CD7-, CD8- by flow cytometry; CD30+, granzyme B+, CD20-, TIA 1- by IHC<br>**Mdx:** NGS: *STAT3* p.S614R (c.1840A>C) (VAF:75%); *KMT2A* (MLL1) p.G20S (c.58G>A) (VAF:33%); *EPHA3* p.S229P (c.685T>C) (VAF:23%); *MALT1* exon5 p.L244F (c.732G>T) (VAF:22%)

Abbreviations: yo year old; yrs years; mo months; LN: lymph node; Morph morphology; IP immunophenotype; + positive; - negative; pT Pathological Tumor stage; N1 regional lymph nodes; N2 Non-regional lymph nodes FISH fluorescence in situ hybridization; rea rearrangement; Mdx molecular analyses; TRG T-cell receptor gamma gene; TRB T-cell receptor beta gene; NGS next generation sequencing; FU: follow up; CHOP cyclophosphamide doxorubicin hydrochloride vincristine sulfate prednisone; PET positron emission tomography; VAF variant allele frequency; VUS variant of unknown significance.
